# Fgl2-knockout tumor cells serve as a vaccine inducing long-duration brain-resident memory T cells that reject subsequent intracranial tumor cell challenges

**DOI:** 10.1016/j.canlet.2025.218215

**Published:** 2025-12-09

**Authors:** Sheng Zhang, Yining Jin, Zhiliang Jia, Xueqing Xia, Yang Li, Qi Wang, Jing Wang, Jian Wang, Joya Chandra, Gregory K. Friedman, Shulin Li

**Affiliations:** a Department of Pediatrics Research, The University of Texas MD Anderson Cancer Center, USA; b Department of Bioinformatics & Computational Biology, The University of Texas MD Anderson Cancer Center, USA; c Department of Biostatistics, The University of Texas MD Anderson Cancer Center, USA

**Keywords:** Brain tumors, Immunotherapy, Resident memory T cells, Fibrinogen-like protein 2, CD47

## Abstract

The failure to prevent brain tumors, including both recurrent primary and metastatic brain tumors, is the primary cause of patients’ mortality. We developed a novel whole tumor-cell vaccine to rapidly induce long-duration brain-resident memory T (T_RM_) cells that prevent brain tumor progression. Ten Fgl2-KO primary and metastatic tumor cell lines, generated via CRISPR/Cas9, were used to vaccinate mice and for intracranial challenges with the WT tumor cells. Not only did vaccinated mice reject these tumor cell challenges, but also more than half of these mice became long-duration survivors. Transplanting brain immune cells from vaccinated mice into naïve mice enabled this rejection of intracranial challenges in the recipient mice, whereas depleting T_RM_ cells impaired it. Mechanistic studies uncovered that Fgl2 KO impaired the immunosuppressive molecule CD47; reconstitution of CD47 expression in Fgl2-KO tumor cells reversed the protection. Likewise, vaccination with CD47-knockdown tumor cells produced similar effects. Proteomic analysis found that Fgl2-KO–mediated suppression of CD47 occurred through the Src and PKCα pathways; inhibition of either pathway reduced CD47 expression. This study is the first to show that disrupting the Fgl2-CD47 circuit in tumor cells impairs their tumorigenic properties and induces long-term brain T_RM_ cells, thereby providing new strategies for improving the efficacy of currently used whole tumor-cell vaccines.

## 1. Background

Brain tumors, including primary tumors (such as glioblastoma) and brain metastases from extracranial tumors, are highly aggressive and make a notable contribution to global morbidity and mortality [1,2]. The 5-year survival rate for patients with primary glioblastoma after standard-of-care treatment is less than 10 % [3], and relapsed glioblastoma as well as metastatic brain tumors are uniformly fatal. Therefore, effective strategies to prevent relapse and metastasis are urgently needed to reduce mortality and improve outcomes for these patients. While many different treatments for relapsed glioblastoma and metastasis have been proposed and studied [4,5], they have been largely ineffective due to the blood-brain barrier, changes in the genetic and epigenetic landscape of tumors, the complexity of neural tissues, and the immunosuppressive brain tumor microenvironment, all of which confer resistance to both standard and emerging therapies [6–9]. Moreover, relatively few strategies to prevent glioblastoma relapse in patients in remission and metastasis to the brain from other tumor sites have been investigated. Such treatments will be essential to reducing the rates of relapse and, therefore, mortality.

One promising strategy to prevent tumor relapse and metastasis is immunotherapy such as vaccination. In two studies, standard care plus dendritic cell (DC)-based vaccines, a hybrid therapy strategy to treat newly diagnosed or relapsed glioblastoma, achieved 3-year overall survival rates of 10 %–17 % [10,11]. A recent phase I/II study found a 24 % 5-year overall survival rate with a DC-based vaccine in patients with newly diagnosed glioblastoma [12]. However, no firm conclusions can be drawn from these studies on treating or preventing relapse or metastasis, which are the primary contributors to patient mortality.

Induction of long-term tumor-specific memory T cells is one possible approach for achieving durable responses and long-term survival for patients with glioblastoma or brain-metastatic solid tumors. In theory, tumor-specific memory T cells can perform immune surveillance against residual disease after standard-of-care treatment. Resident memory T (T_RM_) cells, a long-lived type of memory T cells, have not yet been considered for preventing brain tumor relapse [13] or brain metastasis; however, T_RM_ cells in the skin have been shown to live for years to protect the skin from recurrence of tumors such as melanoma and infections such as herpes simplex virus [14,15]. Brain T_RM_ cells have been observed in aging-associated diseases but have not been investigated for use to prevent brain tumor recurrence.

We recently found that an intravenously administered fibrinogen-like protein 2 (FGL2)-blocking T-cell therapy not only eliminated brain tumors in DBT and GL261 tumor-bearing mice but also induced brain T_RM_ cells to reject subsequent intracranial tumor cell challenge. Interestingly, the FGL2-blocking T cells did not reject subcutaneously implanted tumors, suggesting that the T_RM_ cells were resident in the brain [16]. Indeed, transferring these brain T_RM_ cells to the brains of either immune-competent mice or severe combined immunodeficiency (SCID) mice, which are T cell–deficient, yielded the same results, but depleting the T_RM_ cells by using a CD69-blocking antibody impaired the T_RM_-induced protection from the intracranial tumor cell challenge. We developed this FGL2-blocking T-cell therapy because soluble FGL2 has multiple functions in immune suppression, such as induction of immune checkpoint molecules and immunosuppressive cells including regulatory T cells, myeloid-derived suppressor cells, and M2 macrophages [17]. In addition, FGL2 is enriched in a subpopulation of glioblastoma cells from primary tumors, and Fgl2 knockout (Fgl2 KO) in tumor cells impaired tumorigenesis and tumor progression after intracranial inoculation of tumor cells [18]. However, this approach only works in a few highly immunogenic tumor cell lines.

Based on these data, we tested a subcutaneous Fgl2-KO tumor cell vaccine to induce T_RM_ cells in the brain, which we hypothesized would reject an intracranial tumor cell challenge in murine models. Our central aim was to discover a treatment that can induce T_RM_ cells in mice without brain tumors to model patients whose disease is in remission. We found that subcutaneous Fgl2-KO tumor cell vaccination in naïve (i.e., non–brain tumor-bearing) mice induced T_RM_ cells in brains and resulted in rejection of subsequent intracranial challenges across multiple tumor types, including models of glioblastoma and metastasis from extracranial tumors. Mechanistically, we found a novel molecular circuit between Fgl2 and the “don’t eat me” signaling molecule CD47—Fgl2 induces CD47 via regulating the PKCα and SRC pathways in tumor cells.

## 2. Materials and methods

### 2.1. Animals and cells

Balb/c, C57BL/6, and C3H/HeJ mice were purchased from The Jackson Laboratory (Bar Harbor, ME) and SCID mice from Taconic Biosciences (Germantown, NY). We performed all animal experiments in accordance with the guidelines approved by the Institutional Animal Care and Use Committee (IACUC) at The University of Texas MD Anderson Cancer Center.

DBT cells were kindly provided by Dr. Leonid Metelitsa. GL261 cells were obtained from NCI. Fgl2-KO-DBT, -GL261, -LLC, and -GL261-OVA cells were constructed previously [18]. Fgl2-KO-CT26, -4T1, -K7M3, -B16F10, -LM8, -Ba/F, and –32D cells as well as CD47-related lentivirus were constructed by the VectorBuilder laboratory. The source mouse strains for each cell line are listed in Supplementary Table S1. All cells were cultured as described previously [16,18,19] and treated with a mycoplasma removal agent before experiments. The RetroNectin lentivirus transduction was conducted according to the manufacturer’s protocol.

### 2.2. Mouse models

The tumor mouse models were generated as described previously [16,18,19]. For the subcutaneous tumor model, 1 × 10^6^ tumor cells were suspended in 100 μL PBS and injected subcutaneously into 1 flank of each mouse. For the orthotopic challenge mouse models, unless otherwise indicated, 1 × 10^5^ wild-type tumor cells in a total volume of 5 μL PBS were injected intracranially into Balb/c, C57BL/6, and C3H/HeJ mice. For co-inoculations (or transplant) with BIL cells and tumor cells, 1 × 10^4^ enriched BILs and 1 × 10^3^ tumor cells were mixed and inoculated intracranially into naïve mice of the same strain as the tumor cells used for the vaccination.

### 2.3. CD8, CD69, CD103 depletion

Anti-CD8α (clone 2.43), anti-CD69 (clone CD69.2.2), anti-CD103 (clone M290) (BioXCell West Lebanon, NH), or their matched IgG isotype was injected at a dose of 150 μg per mouse, 2 days before tumor inoculation and every 4 days thereafter. The antibodies were injected intraperitoneally to deplete or block the targets *in vivo*.

### 2.4. In vitro cytotoxic T lymphocyte assay

Target tumor cells were labeled with 1.5 μM CFSE for 30 min. BILs were isolated from vaccinated mice. Target cells and effector BILs were co-cultured for 12 h at ratios of 5:1 and 1:1. CFSE+ 7AAD + tumor cells were detected by flow cytometry.

### 2.5. In vivo bioluminescence imaging and flow cytometry

On the indicated days after vaccination or implantation, mice were injected intraperitoneally with 150 mg/kg of D-luciferin in PBS. Around 10 min later, the mice were imaged with a charge-coupled device camera (IVIS Lumina X5). For brain tumor immune profiling, BILs were harvested to form single-cell suspensions. Fc receptors were blocked. The antibodies used for flow cytometry are listed in Supplementary Table S2. The cell surfaces were stained using a standard flow cytometry protocol.

### 2.6. RPPA and Western blotting

The cell lysates were extracted using RPPA lysis buffer with 4 × sodium dodecyl sulfate (SDS) and sent to MD Anderson’s RPPA Core Facility. Samples were grouped by datasets into separate reports, normalized for loading and set differences, and then median-centered for heatmaps. Western blotting was performed under standard protocols.

### 2.7. Statistical analysis

All quantitative data are presented as mean ± standard deviation or as otherwise indicated. Differences in the experimental means for flow cytometry data were compared using one-way analysis of variance (ANOVA) or t-tests. Two-way ANOVA was used to analyze tumor volume differences between groups. Normality of data was assessed using the Shapiro-Wilk test. The assumption of homogeneity of variances was assessed using the Levene test. For data for which the underlying assumptions were not met, we removed outliers, used a test accounting for unequal variances (e.g., Welch *t*-test and Welch ANOVA) or used a nonparametric test such as the Wilcoxon rank-sum test or Kruskal-Wallis test. The Tukey multiple comparison test was used for pairwise comparisons in the ANOVA. Survival curves were compared by Kaplan-Meier analysis and log-rank tests. For the comparison of repeated-measure tumor volumes, a robust linear mixed model was employed due to the violation of the normality assumption for residuals. All data shown are representative of 3 independent experiments conducted in triplicate (*in vitro*) or at least 2 independent experiments (*in vivo*). Unless otherwise noted, statistical tests were 2-sided and *P* < 0.05 was considered statistically significant. Statistical analyses were conducted using GraphPad Prism 7 software (GraphPad Software, La Jolla, CA) and R software (R Development Core Team, Version 4.4.1).

## 3. Results

Vaccination with Fgl2-KO tumor cells prevents development of brain tumors after tumor-cell challenge.

Both whole tumor cell–based vaccines and T-cell therapy have been used to treat brain tumors in mouse models and humans, but neither has been investigated for prevention of brain tumor development or relapse [16,20]. Also, existing whole tumor cell–based vaccines from others require introduction of other immune-stimulatory genes into the tumor cells, which raises the risk of adverse effects. Here, we investigated whether subcutaneously inoculating Fgl2-KO tumor cells in naïve immunocompetent mice could prevent brain tumor development. Briefly, Balb/c mice were subcutaneously inoculated on day 0 with Fgl2-KO-DBT murine astrocytoma cells (Vac + group) or phosphate-buffered saline (PBS; Vac− group; control). These Fgl2-KO-DBT tumor cells had lost their *in vivo* tumorigenicity (Supplementary Fig. 1a) although they grow normally *in vitro* [21]. Between 12 and 14 days after inoculation, both groups were intracranially challenged with wild-type DBT cells.

**Fig. 1.**
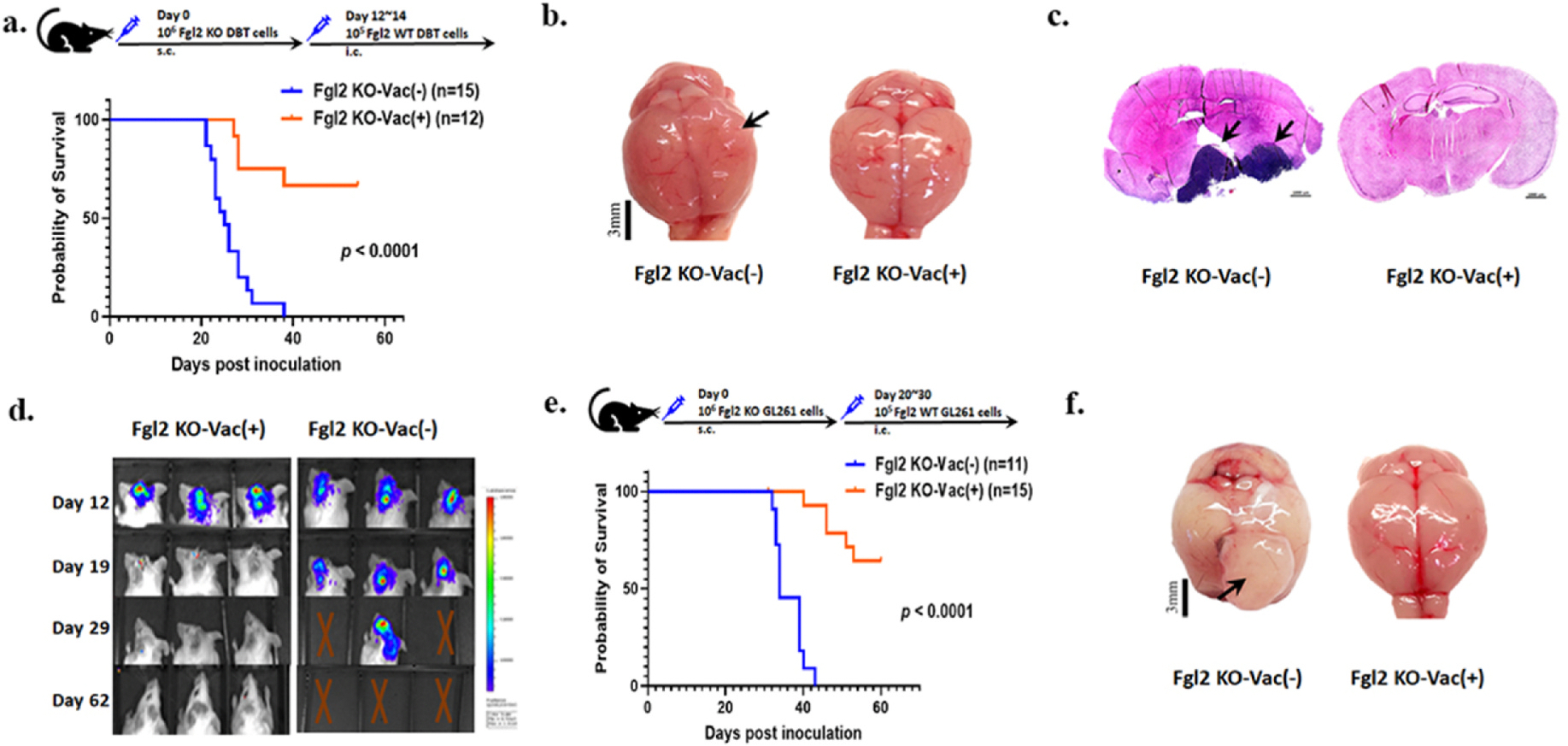
Vaccination with Fgl2 KO tumor cells protects mice from primary brain tumor development **a.** Experimental scheme of the Fgl2 KO DBT whole cell vaccination (top) and Kaplan-Meier survival curves of mice after WT DBT tumor cell challenge (bottom). Balb/c mice were subcutaneously vaccinated with 1 × 10^6^ Fgl2 KO DBT cells (Vac (+) group) or PBS (Vac (−) group) on day 0. Both groups received intracranial injection of 1 × 10^5^ WT DBT cells on day 12. Data are combined from 5 independent experiments with n = 12 for Vac(+) (n = 3, 3, 3, 3, 0) and n = 15 for Vac(−) (n = 3, 3, 3, 3, 3). Log-rank test was performed to compare survival of the 2 groups (MST (median survival time: Vac(−) 25 days vs Vac(+) undefined). **b**. Representative whole-brain pictures on the day of sacrifice of Vac (+) vs. Vac (−) mice after intracranial injection of WT DBT cells. **c**. Comparison of H&E staining of intact mouse brain sections from Vac (+) group vs. Vac (−) group. **d**. Representative bioluminescence (BLI) images of kinetic progress of tumors in the Vac (+) and Vac (−) groups on days 12, 19, 29, and 62. Colored scale bar represents BLI radiance intensity in photons/second/cm^2^/steradian. **e**. Experimental scheme of the Fgl2 KO GL261 whole-cell vaccination (top) and Kaplan-Meier survival curve following WT GL261 tumor cell challenge (bottom). C57BL mice were subcutaneously vaccinated with 1 × 10^6^ Fgl2 KO GL261 cells (Vac (+) group) or PBS (Vac (−) group) on day 0. Both groups received intracranial injection of 1 × 10^5^ WT GL261 cells on day 12. Data are combined from 5 independent experiments with n = 11 for Vac(+) (n = 2, 6, 3, 0,4) and n = 15 for Vac(−) (n = 1, 0, 6, 3, 2). Log-rank test was performed to compare the survival difference of the 2 groups (MST: Vac(−) 34 days vs Vac(+) undefined). **f**. Representative whole-brain pictures on the day of sacrifice of Vac (+) group vs. Vac (−) group after intracranial injection of WT GL261 cells.

About more than 50 % of the Vac + mice did not develop brain tumors after these challenges (Fig. 1a). Examination of whole brains and hematoxylin and eosin (H&E)-stained brain sections confirmed aggressive invasion of DBT tumors in the Vac− group but showed no visible tumor in the long-term (>60 days) survivors from the Vac + group (Fig. 1b and c). One week after the challenge, Vac + mice did not have noticeable signs of brain tumors on bioluminescence imaging. However, tumor growth was evident in the Vac− mice 1 week after the challenge (Fig. 1d).

One prominent and common challenge in treating brain cancer is tumor recurrence after remission, and developing durable immune response holds the key for reducing or eliminating this recurrence. To assess the durability of the Fgl2-KO-DBT vaccine’s protection against tumor recurrence, mice vaccinated with Fgl2-KO-DBT cells underwent multiple challenges after the first challenge using wild-type DBT cells. None of these challenges caused any tumor progression prior to day 80, but multiple mice experienced tumor growth after rechallenge on day 103 (Supplementary Fig. 1b).

To test whether subcutaneous Fgl2-KO tumor cell vaccination could prevent tumor development or relapse in another glioblastoma mouse model in a different mouse strain, immunocompetent C57BL/6 mice were vaccinated with Fgl2-KO-GL261 tumor cells, and wild-type GL261 tumor cells were intracranially injected into mice on days 20–30 after vaccination. Compared with mice in the Vac− control group, mice in the Vac + group had significantly longer median and overall survival times (Fig. 1e). Analysis of whole brains and H&E-stained brain sections confirmed that long-term survivors from the Vac + group showed no signs of brain tumors after the GL261 tumor cell challenge (Fig. 1f, Supplementary Fig. 1c).

### 3.1. Vaccination with subcutaneous Fgl2-KO tumor cells prevents metastatic tumors from forming in the brain after tumor cell challenge

Brain metastases from tumors originating in other organs account for at least half of intracranial tumors in adults, and patients with brain metastases have a median survival duration of less than a year [22]. Brain metastases are most likely to spread from primary lung, breast, and melanoma tumors. Other cancers, including colon cancer, leukemia, sarcoma, gynecologic cancers, and renal cell carcinoma, can also metastasize to the brain [23–25]. To determine whether our subcutaneous Fgl2-KO tumor cell vaccination elicited the same protection against extracranial cancers in the brain as it did against glioblastoma, we generated Fgl2-KO tumor cells from murine CT26 (colon), LLC (lung), K7M3 and LM8 (bone), 4T1 (mammary gland), B16F10 (skin), and 32D and Ba/F3 (bone marrow) cancer cell lines (Supplementary Fig. 2a–d). To avoid any concern that the observed effect could be attributed to viral infection, both viral and nonviral CRISPR/Cas9 methods were used (Supplementary Fig. 2a). We used the same experimental scheme as the subcutaneous Fgl2-KO-DBT vaccine experiment and included Balb/c, C57BL/6, and C3H host mice to correspond to the strain of origin of the tumor cell lines. Mice were intracranially challenged with wild-type tumor cells of the cognate tumor type on day 12.

**Fig. 2.**
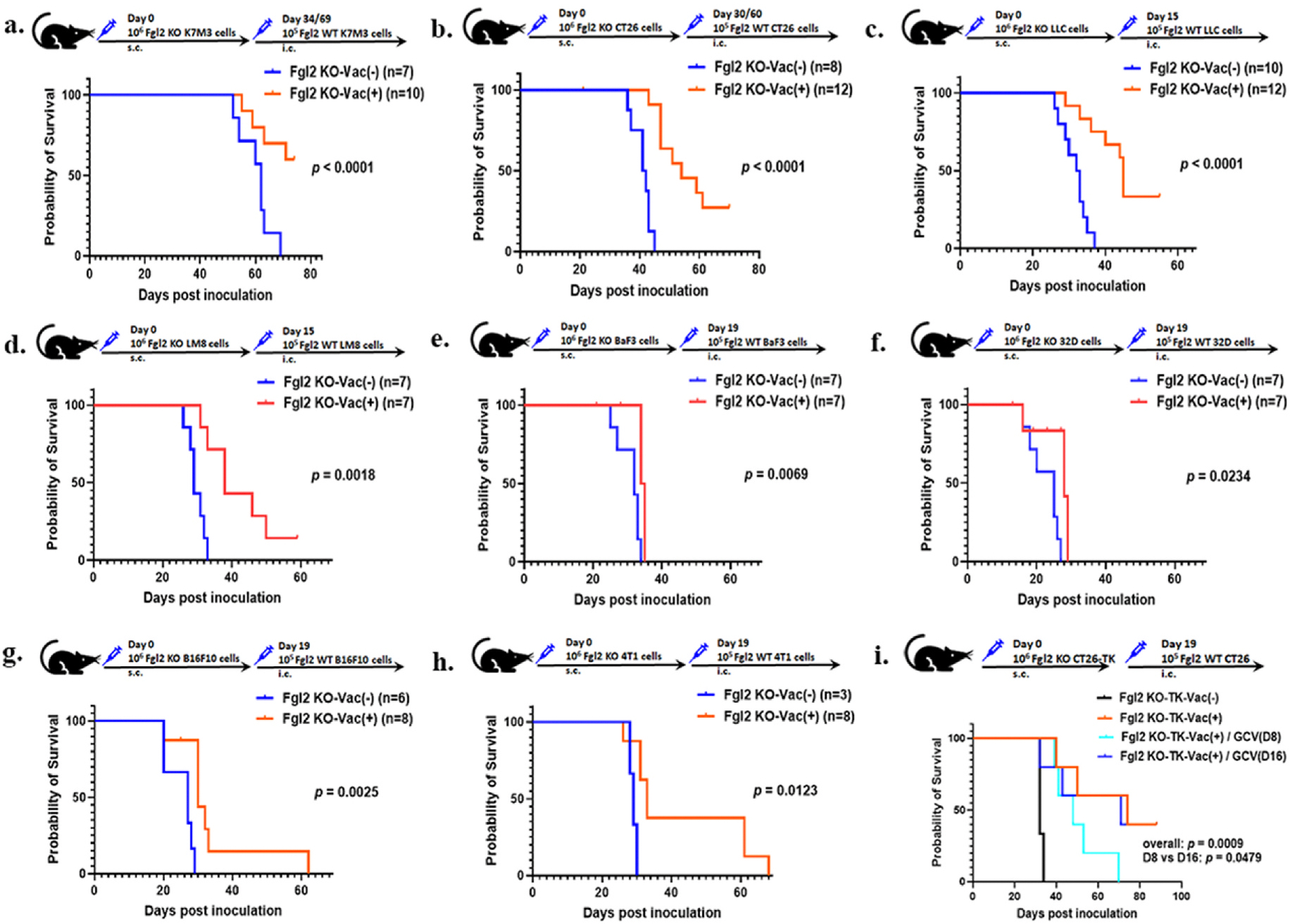
Vaccination with Fgl2 KO tumor cells protects mice from metastatic brain tumor development **a**. Experimental scheme of Fgl2 KO K7M3 whole-cell vaccination (top) and Kaplan-Meier survival curve of mice receiving WT K7M3 tumor cell challenge (bottom). Data are combined from 3 independent experiments with n = 10 for Vac(+) (n = 5, 0, 5) and n = 7 for Vac(−) (n = 0, 3, 4). Log-rank test was performed to compare the survival of the 2 groups (MST: Vac(−) 62.0 days vs Vac(+) undefined). **b**. Experimental scheme of the Fgl2 KO CT26 whole cell vaccination (top) and Kaplan-Meier survival curves of mice receiving CT26 tumor cell challenge (bottom). Data are combined from 4 independent experiments with n = 12 for Vac(+) (n = 3, 9, 0, 0) and n = 5 for Vac(−) (n = 0, 0, 3, 2). Log-rank test was performed to compare the survival of the 2 groups (MST: Vac(−) 41.5 days vs Vac(+) 54.0 days). **c**. Experimental scheme of the Fgl2 KO LLC whole-cell vaccination (top) and Kaplan-Meier survival curves of mice receiving WT LLC tumor cell challenge (bottom). Data are combined from 2 independent experiments with n = 12 for Vac(+) (n = 8, 0) and n = 10 for Vac(−) (n = 4, 10). Log-rank test was performed to compare the survival difference of the 2 groups (MST: Vac(−) 32.5 days vs Vac(+) 45.0 days). **d ~ h**. Experimental scheme of the Fgl2 KO LM8, Baf3, 32D, B16F10, and 4T1 whole cell vaccinations (top) and Kaplan-Meier survival curves of mice receiving WT tumor cell challenge (bottom). Data are combined from 1 (LM8 (MST: Vac(−) 29.0 days vs Vac(+) 38.0 days), 32D (MST: Vac(−) 25.0 days vs Vac(+) 28.0 days), and BaF3 (MST: Vac(−) 32.0 days vs Vac(+) 34.5 days); n = 10 and n = 7 for Vac(+) and Vac(−), respectively) or 2 (B16F10; n = 8 and n = 6 for Vac(+) (MST 30.0 days/n = 3, 5) and Vac(−) (MST 27.0 days/n = 3, 3), respectively; 4T1 (n = 8 and n = 3 for Vac(+) (MST 33.0 days/n = 3, 5) and Vac(−) (MST 29.0 days/n = 0, 3), respectively) independent experiments. Log-rank test was performed to compare the survival of 2 groups. **i**. Experimental scheme of the thymidine kinase (TK)-expressing and Fgl2 KO CT26 whole-cell vaccination (top) and Kaplan-Meier survival curves of mice receiving WT CT26 tumor cell challenge (bottom). Balb/c mice were subcutaneously vaccinated with 1 × 10^6^ TK-expressing and Fgl2 KO CT26 cells (Vac (+) group) or PBS (Vac (−) group) on day 0. On the indicated days, 2 Vac (+) groups received 2 doses (spaced 5 days apart) of GCV. Both groups received intracranial injection of 1 × 10^5^ WT CT26 cells on day 12. Data were collected from 1 of the repeated independent experiments with n = 5 in each group. Log-rank test was performed to compare the survival of different groups (MST: Vac(−) 32.0 days; Vac(+) 74.0 days; Vac(+)/GCV D8 48.0 days; Vac(+)/GCV D16 71.0 days).

Survival analysis revealed 2 distinct groups. Mice vaccinated with Fgl2-KO-K7M3, Fgl2-KO-CT26, Fgl2-KO-LLC, and Fgl2-KO-LM8 cells were protected from challenge with wild-type tumor cells with some long-term survivors (Fig. 2a–d). Visualization of intact mouse brains showed invasion of wild-type tumors in the brains of the Vac− control mice but not in the vaccinated long-term survivors (Supplementary Fig. 2e–g). For the other models, Fgl2-KO-Ba/F3, Fgl2-KO-32D, Fgl2-KO-B16F10, and Fgl2-KO-4T1, there were no long-term survivors among Fgl2-KO–vaccinated mice (Fig. 2e–h). In the 4T1 tumor model, Fgl2-KO–vaccinated mice had significantly longer median survival compared to control mice (*p* = 0.0123). No mice in any of these 4 models remained tumor-free (Fig. 2h). In summary, among the 8 tumor models tested, only 4 had long-term survivors that remained tumor-free after vaccination with Fgl2-KO tumor cells. This result was not associated with mice strain or sex but was solely dependent on the tumor model used.

To investigate the mechanism underlying long-term tumor-free survival after Fgl2-KO tumor cell vaccination, we designated the Fgl2-KO models that had tumor-free outcomes (DBT, GL261, CT26, K7M3, LLC, and LM8) as the response type 1 (RT1) group and those in which Fgl2-KO tumor cell vaccination did not prevent tumor growth (Ba/F3, 32D, B16F10, and 4T1) in brains as the RT2 group. Visually, the RT1 and RT2 groups differed in the appearance of the subcutaneous vaccine sites. The Fgl2-KO tumor cells in the RT1 group did not form any tumors or formed only benign tumor nodules, while the cognate wild-type tumor cells all formed rapidly progressing tumors at the vaccination site (Supplementary Figs. 1a, 2h, 2i). The Fgl2-KO tumor cells behaved similarly to the cognate wild-type tumor cells in the RT2 group, forming rapidly progressing tumors (Supplementary Fig. 2l).

Although the RT1 cell lines did not form any aggressive tumors and served as an effective tumor vaccine via single injection, one still could raise the concern of safety in using live tumor cells. To address this concern, we tested a suicide whole-cell vaccine by engineering Fgl2-KO cells with the herpes simplex virus thymidine kinase gene (*HSV-tk*), which causes rapid tumor cell death upon treatment with thymidine kinase (TK) substrate and has been used in clinical settings [26]. We engineered Fgl2-KO-CT26 colon cancer cells to overexpress the TK protein, which allowed them to be eliminated by treatment with the suicide substrate of the TK enzyme—the nucleoside analog prodrug ganciclovir. Stable CRISPR/Cas9-edited Fgl2-KO-TK-CT26 tumor cells (Vac+) were subcutaneously inoculated into Balb/c mice on day 0. Ganciclovir or PBS treatments were administered to the mice on days 8 and 16 (Fig. 2i). All groups were intracranially challenged with wild-type CT26 tumor cells on day 19. Vac + mice had significantly longer survival than did Vac− mice (Fig. 2i). Earlier administration of ganciclovir (day 8) was less protective against tumor growth than was later administration (day 16); there were no long-term survivors among mice in the early-ganciclovir group (Fig. 2i). These findings show that ganciclovir effectively eliminated Fgl2-KO-TK-CT26 cells and suggest that ~2 weeks’ duration of vaccination is needed to achieve the maximum protection against the tumor cell challenge.

To determine the duration of T_RM_ function in brains after vaccination, we delayed the first challenge from day 14 to day 29 for the CT26 model and to day 46 for the LLC model (Supplementary Fig. 2j and 2k). In both models, there were long-term survivors that survived multiple rounds of challenges with wild-type tumor cells, up to 150 days after the initial vaccination.

### 3.2. Tumor-specific brain T_RM_ cells were functional in naïve recipient mice following transplant of brain CD45^+^ immune cells from RT1-cell vaccinated mice

**Fig. 3.**
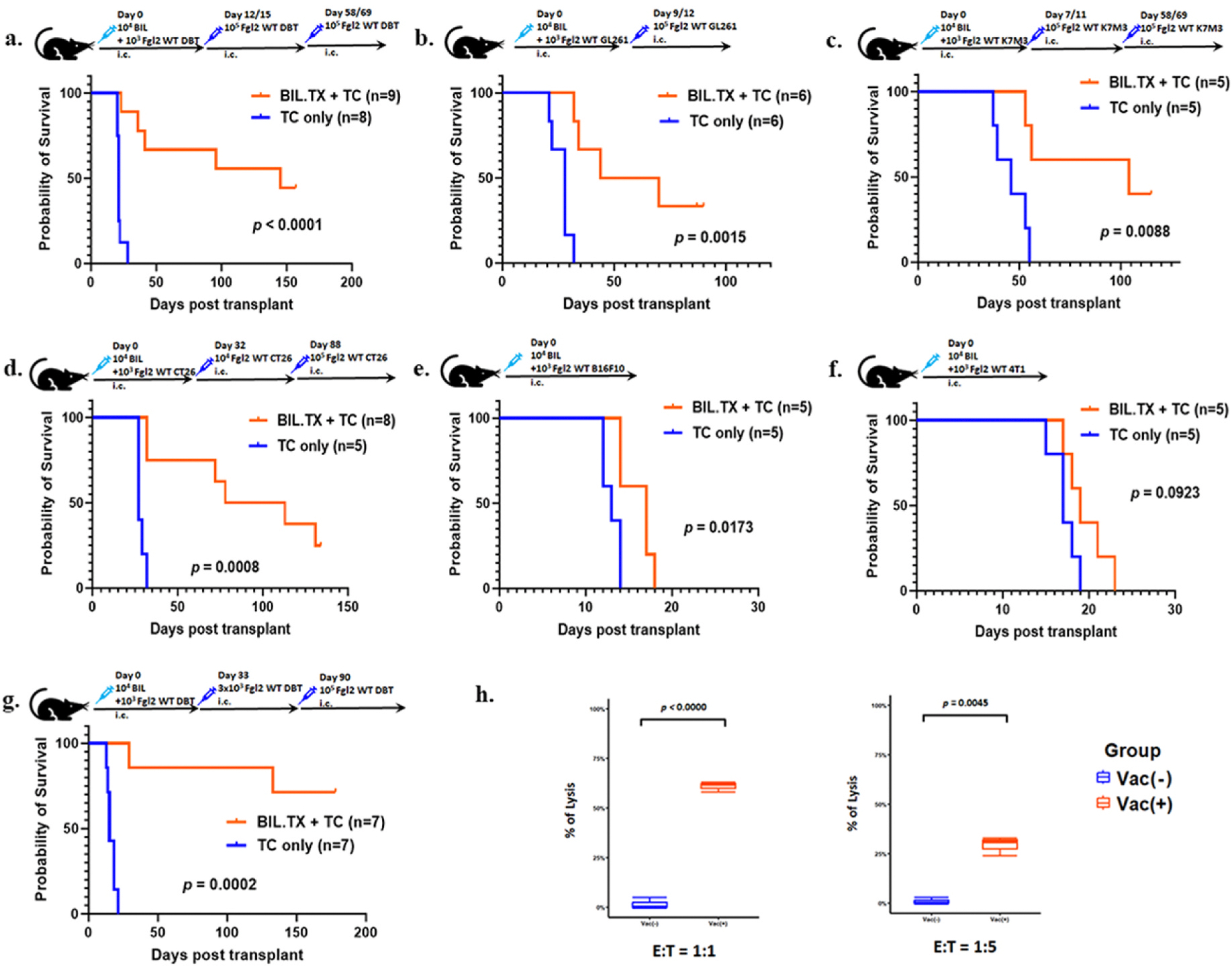
Adoptive transfer of BILs from the Fgl2 KO tumor cell-vaccinated mice **a ~ d**. Experimental scheme of vaccination with Fgl2 KO tumor cells, BIL transplant, and WT tumor cell challenge study (top) and Kaplan–Meier survival curves of mice receiving transplant of BILs and tumor cell challenge (BIL + TC) versus tumor cell challenge only (TC, bottom). Mice were vaccinated subcutaneously with 1 × 10^6^ Fgl2 KO DBT, GL261, K7M3, or CT26 cells. After 10 days, the vaccinated mice were intracranially challenged with the cognate WT tumor cells. After the mice were euthanized and the brains collected, the BILs were harvested and enriched. 1 × 10^4^ BILs with 1 × 10^3^ WT tumor cells or PBS were transferred into the brains of naïve mice. On the indicated days, the transplanted mice were intracranially challenged with 1 × 10^5^ WT tumor cells. Log-rank test was performed to compare survival of 2 groups. Data shown are representative of 2 independent experiments. **a.** DBT (MST 145.0 days/n = 9 for BIL + TC [n = 6, 3] and MST 21.0 days/n = 8 for TC only [n = 5, 3]). **b.** GL261 (MST 57.0 days/n = 6 for BIL + TC [n = 3, 3] and MST 28.0 days/n = 6 for TC only [n = 3, 3]). **c.** K7M3 (MST 104.0 days/n = 5 for BIL + TC [n = 3, 2] and MST 46.0 days/n = 5 for TC only [n = 3, 2]). **d.** CT26 (MST 95.5 days/n = 8 for BIL + TC [n = 3, 5] and MST 27.0 days/n = 5 for TC only [n = 3, 2]). **e ~ f**. Experimental scheme of vaccination with Fgl2 KO tumor cells, BIL transplant, and WT tumor cell challenge study (top) and Kaplan–Meier survival curves of mice receiving transplants of BIL cells plus tumor cell challenge (BIL + TC) versus tumor cell challenge only (TC; bottom). The 1x10^6^ Fgl2 KO B16F10 and 4T1 cells were vaccinated on mice via subcutaneous route. After 10 days, the vaccinated mice were intracranially challenged with the cognate wild type tumor cells. After the mice were euthanized and the brains collected, the BILs were harvested and enriched from brains. 1 × 10^4^ BILs or PBS with 1.5 or 1 × 10^3^ WT tumor cells were transferred into the brains of naïve mice. Log-rank test was performed to compare the survival of 2 groups. **e.** B16F10 (MST 17.0 days/n = 5 for BIL + TC and MST 13.0 days/n = 5 for TC only) **f.** 4T1 (MST 19.0 days/n = 5 for BIL + TC and MST 17.0 days/n = 5 for TC only) **g**. Experimental scheme (top) of vaccination with Fgl2 KO DBT cells, BIL transplant, and WT tumor cell challenge study and Kaplan-Meier survival curves of mice receiving transplants of BILs plus tumor cell challenge (BIL + TC (MST undefined)) versus tumor cell challenge only (TC (MST 15.0 days); bottom). 1 × 10^4^ BILs or PBS with 1 × 10^3^ WT tumor cells were transferred into mouse brains. After 30 and 90 days, 3 × 10^3^ or 1 × 10^5^ WT tumor cells were intracranially injected. **h**. In vitro BIL cytolytic assay. WT GL261 cells were labeled with Calcein AM as target cells. The effector BILs and target GL261-Calcein tumor cells were cocultured at E:T ratios of 1:1 and 1:5 for 12 h. Calcein + 7AAD-positive tumor cells and CD45^+^ BILs were detected by flow cytometry. Data are presented as the mean ± standard error of the mean of triplicate co-culture samples from BILs of primed and control mice from each group.

Our previous study found that intravenous infusion of FGL2-blocking T-cells not only eliminated brain tumors but also induced T_RM_ cells in brains [16]. Hence, we hypothesized that subcutaneous vaccination with Fgl2-KO tumor cells would induce T_RM_ cells in the brain to protect from the observed intracranial challenges described above. To test this hypothesis, we collected CD45^+^ cells (brain-infiltrating leukocytes, BILs), from the brains of vaccinated mice on day 4 following intracranial challenge. We then transplanted either the isolated BILs mixed with wild-type DBT tumor cells or wild-type DBT tumor cells alone into recipient mice of the same mouse strain. All recipient mice were then intracranially challenged with 1 × 10^5^ wild-type tumor cells on days 12~15 (DBT), 9–12 (GL261), 7–11 (K7M3), and 32 (CT26). Mice that received BILs had longer median survival, with several long-term survivors, compared to mice that received only wild-type tumor cells (Fig. 3a). Similar results were observed in the mice that received BILs from mice vaccinated with the RT1 tumor cells Fgl2-KO-GL261, Fgl2-KO-K7M3, and Fgl2-KO-CT26 (Fig. 3b–d). As expected, the mice that were vaccinated with the RT2 cell lines B16F10-Fgl2-KO and 4T1-Fgl2-KO showed no protection against intracranial tumor cell challenge (Fig. 3e–f).

To confirm that this observation was not caused by inducing a host T-cell response in the recipient mice, we transplanted BILs from mice that had been vaccinated with Fgl2-KO-DBT cells into the brains of T cell–deficient naïve SCID mice. We found that transplantation induced similar protection against the intracranial DBT challenge as in the immunocompetent mice (Fig. 3g). To verify the antitumor function of the BILs in the RT1 group, we measured their cytotoxic activity *in vitro*. BILs were isolated from DBT-vaccinated mice or control mice and used as effector cells. Wild-type DBT tumor cells were labeled with carboxyfluorescein succinimidyl ester (CFSE) as target cells. Target cells and effector cells were co-cultured at ratios of 5:1 and 1:1 for 12 h, and CFSE+ 7AAD-positive live tumor cells were detected by flow cytometry. As expected, over 60 % of tumor cells were killed after coculture with the Vac + BILs and nearly none with the Vac− BILs at an effector-to-target ratio of 1:1 (61 % versus 2 %, respectively), which demonstrated the antitumor activity of these BILs (Fig. 3h).

To validate that these T_RM_ cells truly derived from the brain and not from splenocytes that had migrated from the spleen, we transplanted splenocytes into the brains or peripheral tissues of recipient mice. A low number of RT1-vaccinated mice that received transplants of splenocytes survived long-term, and the number of splenocytes was 100 times that of BILs, which provided better long-term survival (Supplementary Fig. 3a–3d). These findings demonstrate that some splenocytes can become T_RM_ cells that reject brain tumor cell challenge, though at a low frequency. In support of this observation, intravenous adoptive transfer of splenocytes from the RT1 cell–vaccinated mice also yielded long-term survivors in 2 glioblastoma models (Supplementary Fig. 3a and 3b), showing that T cells from the spleen circulated in the brains of the vaccinated mice to become T_RM_ cells. Again, the success rate was much lower despite transplantation of 100-fold more CD45^+^ cells, suggesting that the rapid rejection of intracranial tumor cell challenges (within days, as shown in Fig. 2d) was dependent on brain T_RM_ cells. As expected, transplanting splenocytes from RT2 cell (B16F10 and 4T1)–vaccinated mice were not successful (Fig. 3e and f).

### 3.3. Phenotypic and functional validation of T_RM_ induction in brains via subcutaneous Fgl2-KO tumor cell vaccination

**Fig. 4.**
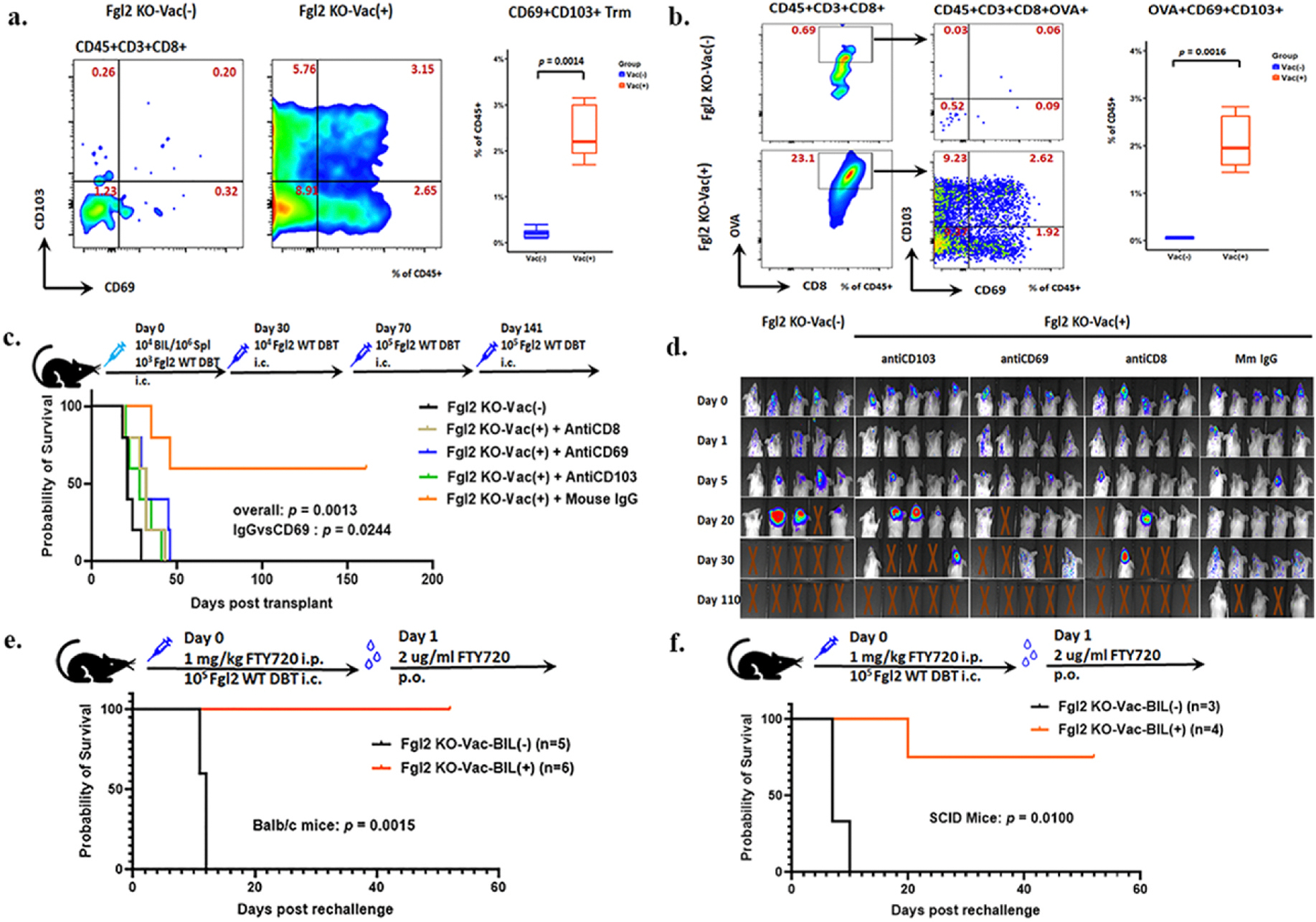
Identification of tissue resident memory (T_RM_) CD8 T cells in the BILs of mice vaccinated with Fgl2 KO tumor cells and their relevance in rejecting intracranial tumor cell challenge **a**. Representative flow cytometry plots showing increase of CD8^+^ CD69^+^ CD103^+^ T cells in mice vaccinated with Fgl2 KO GL261-OVA cells. 1 × 10^6^ Fgl2 KO GL261OVA cells were injected subcutaneously into mice. After 10 days, the vaccinated mice were intracranially challenged with WT GL261-OVA cells. The mice were euthanized on day 4, and the brains were collected. The BILs from recipient mice were harvested and stained with surface immune markers. Percentages of CD8^+^ CD69^+^ CD103^+^ T_RM_ cells among the CD45^+^ BILs in the brains of the challenged mice are shown. Data are mean ± SD, one-way ANOVA with Tukey multiple comparisons test. **b.** Representative flow cytometry plots showing increase of OVA-specific CD8^+^ T_RM_ T cells in mice vaccinated with Fgl2 KO GL261-OVA cells. Percentages of OVA-specific CD8^+^ T cells (left) and OVA-specific CD8^+^ T_RM_ T cells among the CD45^+^ BILs (right) in the brains of the challenged mice are shown. All plots are representative of 2 independent experiments. **c.** Experimental scheme of vaccination with Fgl2 KO DBT cells, BIL transplant, and T_RM_ depletion, and challenge with WT tumor cells (top). Kaplan–Meier survival curves of mice receiving BIL transplant and tumor-cell challenge. 1 × 10^6^ Fgl2 KO DBT cells were injected subcutaneously into mice. On day 14, the vaccinated mice were intracranially challenged with WT DBT cells. Three days later, the mice were euthanized, the brains were collected, and the BILs were harvested and enriched. 1 × 10^4^ BILs or PBS with 1 × 10^3^ WT tumor cells were transferred into the brains of naïve mice. On days 0, 5, 15, 20 post-transplant, CD8, CD69, CD103, and IgG control antibodies (150 μg/mouse, i.p.) were administered. For tumor cell challenge, 1 × 10^4^ or 1 × 10^5^ WT tumor cells were intracranially injected on days 30, 70, and 141 (MST: Vac(−) 21.0 days; Vac(+) + anti-CD8 32 days; Vac(+) + anti-CD69 32 days; Vac(+) + anti-CD103 28 days; Vac(+) + mouse IgG undefined). **d**. Representative bioluminescence (BLI) images of tumors in the transplanted mice with or without T_RM_ depletion on days 0, 1, 5, 20, and 30. Colored scale bar represents BLI radiance intensity in photons/second/cm^2^/steradian. **e ~ f**. Experimental scheme of vaccination of Balb/c and SCID mice with Fgl2 KO tumor cells, BIL transplant, and WT tumor cell challenge with FTY720 treatment (top) and Kaplan–Meier survival curves of mice receiving BIL transplants plus tumor cell challenge (BIL + TC) versus tumor cell challenge only (TC; bottom). 1 × 10^6^ Fgl2 KO DBT cells were injected subcutaneously into mice. After 10 days, the vaccinated mice were intracranially challenged with the cognate WT tumor cells. After the mice were euthanized and the brains collected, the BILs were harvested and enriched. 1 × 10^4^ BILs or PBS with 1 × 10^3^ WT tumor cells were transferred into the brains of naïve Balb/c and SCID mice. The transplanted mice were challenged with 1 × 10^5^ WT tumor cells via intracranial route 3 times. The surviving mice underwent intracranial challenge with FTY720 treatment. Log-rank test was performed to compare the survival of 2 groups. **e.** Balb/c mice (MST 12.0 days/n = 5 for control and MST undefined/n = 6 for transplanted mice). **f.** SCID mice (MST 9.0 days/n = 3 for control and MST undefined/n = 4 for transplanted mice).

To validate T_RM_ induction in the brain following subcutaneous RT1-group Fgl2-KO tumor cell vaccination, we used the Fgl2-KO-GL261 model. As above, mice were vaccinated with Fgl2-KO-GL261 tumor cells on day 0 and received an intracranial injection of wild-type tumor cells on day 10. BILs were isolated from the brain and analyzed by flow cytometry on day 15. We measured the percentage of BILs expressing the classical T_RM_ phenotypes (CD3^+^, CD8^+^, CD69^+^, and CD103+) [27]. The gating strategy for T_RM_ cells is displayed in Supplementary Fig. 4a. The Vac + group displayed a markedly higher percentage of CD3^+^CD8^+^CD69^+^CD103+ T_RM_ cells than did the Vac− group (3.15 % vs. 0.20 % of CD45^+^ cells, respectively) (Fig. 4a, Supplementary Fig. 4b).

To confirm that these T_RM_ cells are tumor-targeted T cells, we subcutaneously vaccinated naïve mice using Fgl2-KO-GL261 cells engineered to express ovalbumin (OVA) and intracranially challenged these mice with wild-type GL261-OVA tumor cells. OVA tetramer staining was used to isolate OVA-specific (tumor-specific) T cells from the isolated BILs. A higher percentage of OVA-specific CD3^+^CD8^+^CD69^+^CD103+ T_RM_ cells was found in Vac + than in Vac− mice (2.62 % vs. 0.06 % of CD45^+^ cells) (Fig. 4a and b, Supplementary Fig. 4c–d).

To functionally validate the role of brain T_RM_ cells induced by Fgl2-KO tumor cell vaccination, we determined whether T_RM_ depletion impaired tumor rejection after vaccination. We transplanted the T_RM_ cells from vaccinated mice to naïve mice and intracranially challenged the mice following the injection of control IgG or T_RM_ depleting antibodies CD8, CD69, or CD103, respectively. Briefly, we vaccinated and challenged donor mice with subcutaneous Fgl2-KO-DBT and intracranial wild-type DBT cells, respectively, and then intracranially transplanted isolated BILs mixed with wild-type DBT tumor cells to naïve recipient mice. The recipient mice then received multiple rounds of anti-CD8, anti-CD69, or anti-CD103 antibodies or nonspecific IgG antibodies (control) to deplete T_RM_ cells (CD103 or CD69) or the entire CD8^+^ T-cell population. We found that neutralizing antibodies targeting either cytotoxic CD8^+^ T cells or T_RM_ cells (CD69 and CD103) impaired protection against intracranial challenge compared to controls (Fig. 4c and d), showing the exclusive dependence on brain Trm cells for tumor rejection. These results demonstrated that T_RM_ cells are a crucial mechanism underlying rejection of intracranial tumor cell challenge after Fgl2-KO tumor cell vaccination.

To confirm that the observed protection in vaccinated mice is mediated by brain-resident T_RM_ cells rather than circulating or migrating T cells, we inhibited lymphocyte migration using FTY720 and subsequently performed an intracranial tumor challenge in Balb/c and SCID mice that received transplanted BILs. Notably, despite FTY720 treatment, transplanted BILs conferred full protection against intracranial tumor challenge (Fig. 4e and f). These findings strongly indicate that brain-resident T_RM_ cells, rather than other host immune cells, play a critical role in mediating protection against intracranial tumor challenge following Fgl2-KO tumor cell vaccination.

### 3.4. Fgl2 KO mediated loss or reduction of CD47 expression in RT1 but not in RT2 tumor cell models

**Fig. 5.**
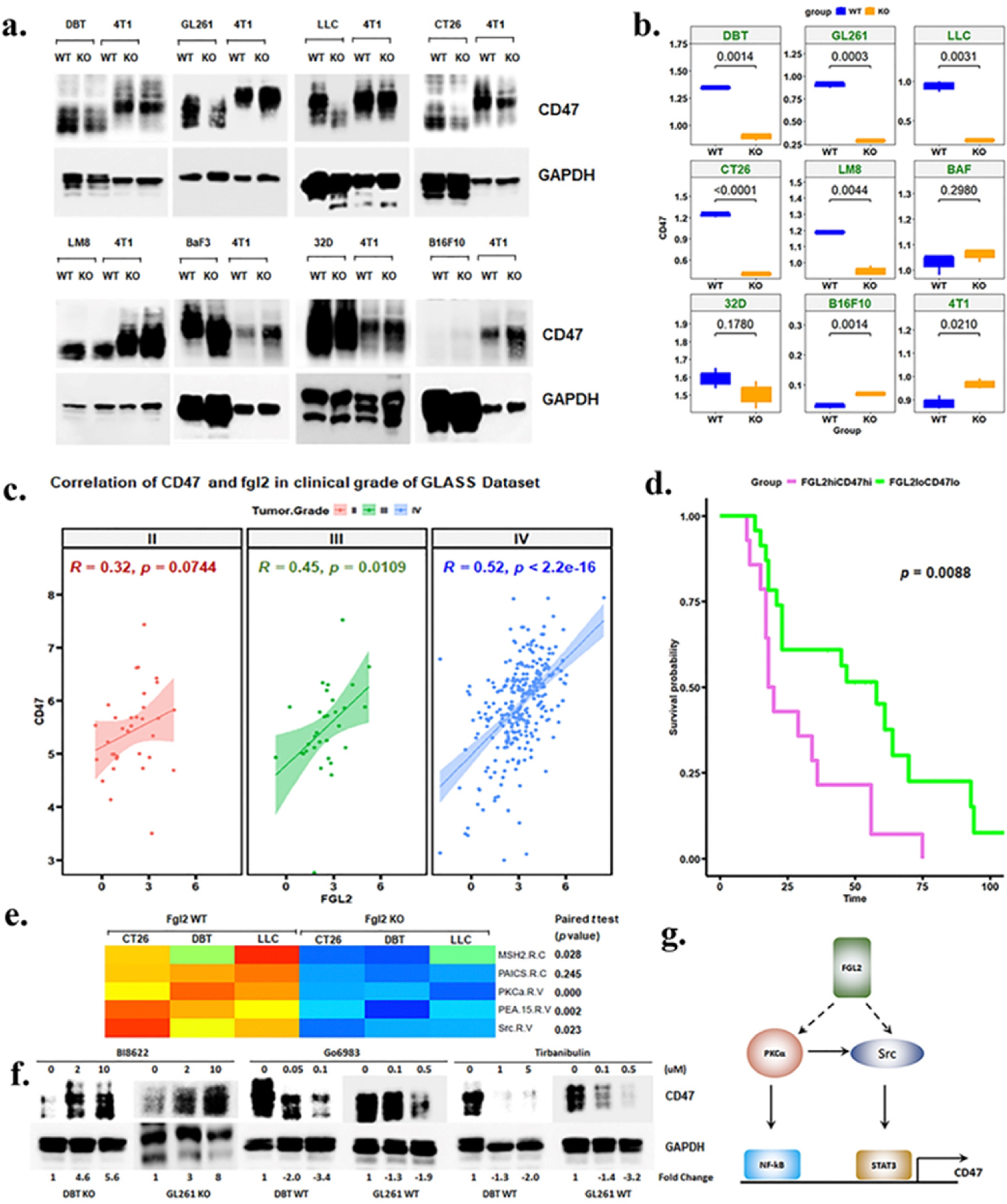
Fgl2 KO in tumor cells is associated with reduction of CD47 expression in RT1 tumor cells **a.** Reduction of CD47 expression in Fgl2 KO tumor cells. Top row: Western blots showing CD47 protein level in each pair of WT versus Fgl2 KO (KO) RT1-type tumor cells. 4T1 cells belong to the RT2 type and served as an internal control. Bottom row: Western blots showing CD47 protein level in each RT2-type tumor cell line pair. **b.** Quantification of CD47 protein levels in different tumor cell pairs. Data are summarized as ratio changes (normalized to GAPDH). The *t*-test was used to calculate two-sided *P* values. **c**. Correlation analysis of mRNA expression levels of FGL2 and CD47 in the Glioma Longitudinal AnalySiS (GLASS) Consortium. The original data were processed by the cBioPortal for Cancer Genomics to z-score (reference to all samples). The data were grouped by the tumor clinical grade (closely linked with patient outcome). R = Pearson correlation coefficient. Data were summarized as ratio changes (normalized to GAPDH). The *t*-test was used to calculate the two-sided *P* values. **d**. Kaplan–Meier overall survival analysis of diffuse glioma patients in the GLASS dataset, grouped by expression levels of FGL2 and CD47 mRNA. Patients’ tumors were classified as FGL2^high^CD47^high^ (>1 SD) and FGL2^low^CD47^low^ (<1 SD). The log-rank test was used to compare overall survival among groups. **e**. Proteomic analysis of Fgl2 WT versus Fgl2 KO RT1 tumor cells. The cell lysates were harvested and subjected to RPPA. Data were normalized to conduct differential expression analysis. The proteins with significantly different relative protein levels (mean net intensities of spots) were selected and are shown by the heatmap. **f**. The effect of Src and PKCα inhibition on CD47 expression. The proteins with the greatest differences on the heatmap in (e) and with relevance to CD47 were chosen. The inhibitors that target the selected proteins were identified from the literature. Tumor cells were treated with different inhibitors against Src and PKCα *in vitro* for 24 h. The expression levels of CD47 were shown by Western blotting in DBT cells and in GL261 cells. **g**. Schematic illustration of cellular and molecular events underlying FGL2-regulated CD47 expression. FGL2 protein activates PKCα and Src as reported in the literature. PKCα simultaneously interacts with Src. PKCα activates the NF-κB pathway, while Src activates the STAT3 pathway. Both NF-κB family proteins and STAT3 bind to the promoter region of the CD47 gene.

To identify the key regulator underlying the T_RM_-mediated rejection of RT1, but not RT2, tumors, we compared the expression levels of multiple key immune-stimulatory or checkpoint proteins in Fgl2-KO tumor cells and their corresponding wild-type tumor cells (Fig. 5a and Supplementary Fig. 5a). We did not explore whether the host strain could account for the difference in T_RM_ induction between the RT1 and RT2 models because we saw differences in T_RM_-induced protection between tumor cell lines derived from the same mouse strain (for example, DBT and 4T1 derive from Balb/c mice). We therefore surmised that a mechanism other than mouse strain was at play. While differences in expression of the candidate proteins between Fgl2-KO and wild-type tumor cells varied within the RT1 and RT2 groups, only the loss or reduction of CD47 consistently occurred in each of the RT1 Fgl2-KO cell models compared to wild-type cell lines, but CD47 levels changed little in the RT2 group cell lines (Fig. 5a and b). Thus, we selected CD47 as the candidate protein that governs the antigenic property of tumor cells. Indeed, studies have shown that the presence of CD47 on the surface of cancer cells allows them to evade immune surveillance [28–30].

To understand the relevance of Fgl2 and CD47 expression for glioblastoma patients, we analyzed RNA sequencing data from a large brain tumor dataset of the Glioma Longitudinal AnalySiS (GLASS) Consortium (329 patients, 693 samples, including partial TCGA data) [31,32]. The original data were processed by the cBioPortal for Cancer Genomics into z-scores [33,34], and we calculated the Pearson correlation coefficient between FGL2 and CD47 expression levels by clinical tumor grade (Fig. 5c, Supplementary Fig. 5b). The correlation between FGL2 and CD47 was more significant as tumor grade increased (*p* = 0.07, 0.01, and 2.2 × 10^−16^ for grade II, III, and IV tumors, respectively).

To further understand the clinical relevance of FGL2 and CD47 for patient outcomes, we performed survival analysis based on FGL2 and CD47 expression in the GLASS dataset [32]. The patients were divided into FGL2-high/CD47-high or FGL2-low/CD47-low groups, which represented expression levels of FGL2 and CD47 that were at least 1 standard deviation higher or lower than the mean expression level of all patients. Patients with high concurrent expression of FGL2 and CD47 had significantly worse survival outcomes compared to patients with low expression (Fig. 5d). This finding suggests that high concurrent expression of FGL2 and CD47 may contribute to more aggressive tumor behavior and poorer patient prognosis.

### 3.5. Fgl2 regulates the signaling transduction circuit of CD47

To evaluate the mechanisms underlying these changes in CD47 expression, we first performed a reverse phase protein array (RPPA) analysis of the RT1-group cell line pairs (Fgl2 wild-type vs Fgl2-KO) to uncover the key protein that impacts Fgl2-mediated CD47 expression (Supplementary Fig. 5c). Three proteins (Lasu1, PKC- α, and Src) that significantly differentiated wild-type from Fgl2-KO cells and may be relevant to CD47 expression were identified (Fig. 5c and e). Lasu1 (Supplementary Fig. 5c), a tumor suppressor that stabilizes TP53 [35], was more highly expressed in Fgl2 KO tumor cells than in wild-type Fgl2 tumor cells, whereas PKC-α (Fig. 5e), a signaling molecule associated with tumor growth [36], was more highly expressed in wild-type tumor cells. We also found that expression levels of Src (Fig. 5e), a tyrosine kinase linked to cancer progression that has been reported to bind and phosphorylate CD47 to evade immune surveillance, as well as its activated phosphorylated form SRC-PY527, were both elevated in the wild-type tumor cells relative to the Fgl2-KO tumor cells [37].

To verify that CD47 is regulated by these 3 proteins, we conducted *in vitro* inhibition studies. Substantially higher CD47 expression was observed in both Fgl2-KO-DBT and Fgl2-KO-GL261 tumor cells after treatment with the Lasu1 inhibitor BI8622 [38] than in the corresponding untreated control cells (Fig. 5f), demonstrating that Fgl2 KO-mediated Lasu1 upregulation (Supplementary Fig. 5c) plays a key role in reducing CD47 expression. Furthermore, in agreement with the RPPA results, treatment of the wild-type tumor cells with either the PKC pan-inhibitor Go6983 [39] or the SRC inhibitor tirbanibulin [40] substantially suppressed the CD47 levels in the cell lysates, showing that SRC and PKC-α are involved in the upregulation of CD47 (Fig. 5f).

Taken together, these results demonstrate that Fgl2 is an upstream regulator that boosts the activity of PKCα and Src. PKCα leads to the activation of NF-κB and Src, which triggers STAT3 activation. NF-κB and STAT3 signaling converge to boost the expression of CD47 (Fig. 5g).

### 3.6. CD47 in tumor cells regulates T_RM_ induction in the brain after subcutaneous Fgl2-KO tumor cell vaccination

**Fig. 6.**
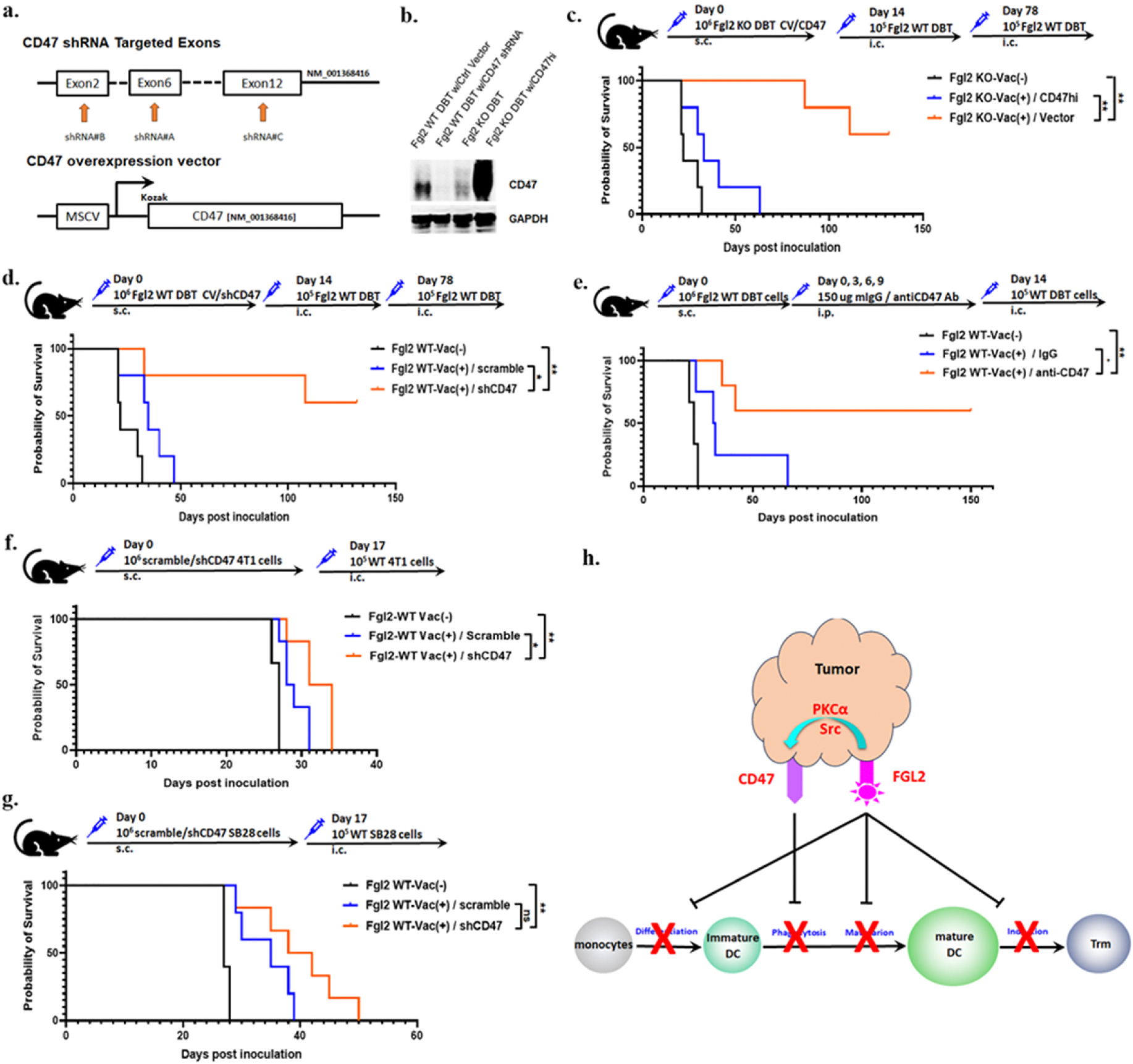
Vaccination with CD47-overexpressing or CD47-silenced tumor cells protects mice from primary brain tumor development **a**. The targeted CD47 exons of CD47 shRNA based on the optimal knockdown scores to achieve highly efficient targeting of genes of interest are shown in a schematic diagram (VectorBuilder.com). **b**. Confirmation of CD47 knockdown or overexpression via Western blotting. The WT DBT cells were transduced with scramble RNA or CD47 shRNA, and Fgl2 KO DBT cells were transduced with control vector or CD47. Transduced cells were treated with 0.06 U/mL bleomycin to obtain a stable cell mixture. **c**. Experimental scheme (top) of CD47-overexpressing Fgl2 KO DBT cell vaccination and survival analysis (bottom). Balb/c mice were subcutaneously vaccinated with 1 × 10^6^ CD47^high^ or control vector Fgl2 KO DBT cells (Vac(+) group) or PBS (Vac (−) group) on day 0. All groups received intracranial injection of 1 × 10^5^ WT DBT cells on day 14. Kaplan-Meier survival analysis was performed across these 3 groups (n = 5). Log-rank test was performed to compare the survival of 2 or 3 groups (MST: Vac(−) 22.0 days; Vac(+)/Vector 33.0 days; and Vac(+)/CD47hi undefined). **d**. Experimental scheme (top) of silenced CD47 Fgl2 WT DBT cell vaccination and survival analysis (bottom). Balb/c mice were subcutaneously vaccinated with 1 × 10^6^ silent CD47 or control vector Fgl2 WT DBT cells (Vac(+) group) or PBS (Vac (−) group) on day 0. All groups received intracranial injection of 1 × 10^5^ WT DBT cells on day 14. Kaplan-Meier survival analysis was performed across these 3 groups (n = 5). Log-rank test was performed to compare the survival of 2 or 3 groups (MST: Vac(−) 22.0 days; Vac(+)/scramble 35.0 days; and Vac (+)/CD47 shRNA undefined). **e**. Experimental scheme (top) of vaccination with Fgl2 WT DBT cells and administration of a CD47-neutralizing antibody and survival analysis (bottom). Balb/c mice were subcutaneously vaccinated with 1 × 10^6^ Fgl2 WT DBT cells (Vac(+) group) or PBS (Vac (−) group) on day 0. The isotype control IgG and anti-CD47 antibodies (150 μg per mouse) were administrated i.p. on days 0, 3, 6, and 9. All groups received intracranial injection of 1 × 10^5^ WT DBT cells on day 14. Kaplan-Meier survival analysis was performed across these 3 groups (n = 4, n = 3, and n = 5 for control, IgG, and anti-CD47 antibody, respectively). Log-rank test was performed to compare the survival of 2 or 3 groups (MST: Vac(−) 23.0 days; Vac(+)/IgG 32.5 days; and Vac(+)/anti-CD47 undefined). **f ~ g**. Experimental scheme (top) of CD47 silencing in vaccination with Fgl2 WT 4T1 or SB28 cells and survival analysis (bottom). Balb/c (4T1 cells) and C57BL/6 (SB28 cells) mice were subcutaneously vaccinated with 1 × 10^6^ silent-CD47 or control vector Fgl2 WT tumor cells (Vac (+) group) or PBS (Vac (−) group) on day 0. All groups received intracranial injection of 1 × 10^5^ WT tumor cells on day 14. Kaplan-Meier survival analysis was performed across the 3 groups (n = 5). Log-rank test was performed to compare the survival of 2 or 3 groups. **f**. 4T1 cells (MST: Vac(−) 27.0 days; Vac(+)/scramble 28.5 days; and Vac(+)/CD47 shRNA 32.5 days). **g**. SB28 cells (MST: Vac (−) 27.0 days; Vac(+)/scramble 35.0 days; and Vac(+)/CD47 shRNA 40.0 days). **h**. Schematic illustration of multiple tumor immunosuppressive mechanisms in which Fgl2 and CD47 synergistically modulate tumor immunogenicity.

CD47 is widely recognized for its role in promoting tumor invasion and metastasis [29], and various strategies targeting CD47 have been employed in cancer immunotherapy [41–43]. Our results above uncovered a novel molecular circuit: KO of Fgl2 significantly reduced CD47 levels in RT1 tumor cell lines, inducing tumor-specific T_RM_ cells in the brain (Figs. 1, 2 and 5). To validate this discovery, we reconstituted CD47 expression in the Fgl2-KO-DBT cell line and knocked down CD47 expression in wild-type DBT tumor cells using CD47 cDNA or shRNA vectors, respectively (Fig. 6a). Western blot analysis confirmed that CD47 was reconstituted and overexpressed in the Fgl2-KO-DBT clone (Fig. 6b, last lane). Next, we tested whether CD47 reconstitution abolishes T_RM_ induction by vaccination. As expected, vaccination with the CD47-reconstituted Fgl2-KO-DBT vaccine failed to induce the same immune protection as Fgl2-KO-DBT vaccination (Fig. 6c).

Next, we generated a CD47-knockdown DBT cell line using a CD47 shRNA (shCD47) and wild-type DBT cells (Fig. 6a). Wild-type DBT cells were used because Fgl2 is present in this cell line, as we previously demonstrated [16], and inoculation of these wild-type tumor cells in mice led to rapid tumor formation and growth [21]. To determine if shutting down CD47 alone in wild-type DBT cells is sufficient to induce T_RM_ cells, we first validated CD47 downregulation via Western blotting (Fig. 6b) before vaccinating mice via subcutaneous injection of shCD47-DBT cells on day 0, and then we performed a challenge with wild-type tumor cells on day 14 after vaccination. As expected, 80 % of mice survived the intracranial wild-type tumor cell challenge for more than 100 days (Fig. 6d), suggesting that the reduction of CD47 following Fgl2 KO has a crucial role in inducing immune protection against intracranial tumor cell challenge.

To investigate whether blocking CD47 function in wild-type DBT cells using a CD47-neutralizing antibody confers protection against intracranial tumor challenge, we administered anti-CD47 antibodies to naïve mice during vaccination. Specifically, naïve mice were subcutaneously vaccinated with Fgl2-WT-DBT cells and subsequently received multiple doses of either anti-CD47 antibodies or nonspecific IgG antibodies (control) to inhibit CD47 function in tumor cells. Fourteen days after vaccination, the mice underwent intracranial challenge with wild-type tumor cells. As anticipated, 60 % of the mice survived beyond 30 days following the challenge (Fig. 6e), further supporting the role of CD47 blockade in eliciting immune protection against intracranial tumor cell challenge.

To further explore the role of CD47 in highly malignant tumor cells, we generated 2 additional CD47-knockdown cell lines, SB28 and 4T1, using CD47-targeting shRNA (shCD47) and scramble RNA as a control. Naïve mice were vaccinated via subcutaneous injection of either shCD47-or scramble RNA-expressing tumor cells on day 0 and were challenged with wild-type tumor cells on day 14. While a statistically significant difference in survival time was observed between the shCD47 and scramble control groups (Fig. 6f and g), no mice survived in either cohort. These findings suggest that in SB28 and 4T1 cells, additional unidentified mechanisms may counteract the immune protection conferred by CD47 reduction, ultimately impairing the provoked immune response against intracranial tumor challenge.

## 4. Discussion

Brain tumors are broadly categorized into two types: primary brain tumors such as gliomas and metastatic brain tumors, which occur approximately 4 times more frequently than primary brain tumors. Despite advances in targeted therapy and immunotherapy for brain tumors, the long-term survival rate for patients with recurrent glioblastoma or brain metastases from extracranial sites is approximately 5 % [2]. Thus, prevention of tumor relapse and metastatic tumors in the brain would likely improve survival outcomes [6]. Whole tumor cell-based vaccines (WCV) represent one approach, but WCV often requires an insertion or overexpression of immune stimulatory genes such as CSF that unfortunately cause side effects. Also, tumors continue to grow after modified WCV administration, and therefore, tumor cell irradiation is required for clinical practice. Here, we developed a novel WCV—Fgl2 KO tumor cell vaccine that does not require any gene insertion in tumor cells. To facilitate safety, a suicide system was tested, and we confirmed that this suicide gene-armed Fgl2KO WCV works as expected. Of course, if concerns about adverse effects arise, we could also use the classical irradiation technique to generate the Fgl2KO WCV.

We used Fgl2KO to serve as a WCV in this study because Fgl2 is highly expressed in a subset of cells in gliomas. High expression of FGL2 is associated with tumor progression and poor survival in several cancers, including glioblastoma [18]. FGL2 secreted from tumor cells was found to suppress antigen-presenting CD103+ dendritic cells that induce tumor-specific T-cell responses. Furthermore, we previously showed that intracranial inoculation with Fgl2-KO tumor cells inhibits tumor formation in immunocompetent mice and that this inhibition is reversed when CD103 is disrupted, making FGL2 a promising therapeutic target [18].

FGL2-blocking antibodies have been tested in several tumor-bearing mouse models and significantly prolonged survival in both glioma and hepatocellular carcinoma models [44,45]. Previously, we found that administration of an oligoclonal anti-FGL2 antibody induced antitumor activity and extended the survival time of GL261 glioma-bearing mice; similar effects were observed in melanoma, Lewis lung carcinoma, and astrocytoma mouse models [16,21]. However, anti-FGL2 antibodies only resulted in prolonged survival in these models and did not prevent brain tumor recurrence in mice in remission [16]. The failure of the anti-FGL2 antibody therapy to reliably prevent brain tumor relapse may have been due to the limited ability of the antibody to penetrate the blood-brain barrier or the low functional activity of the monoclonal antibody; a polyclonal FGL2 antibody has been shown to be more effective *in vivo* [16,21].

To address some of these concerns, T cells engineered with an FGL2 single chain variable fragment (FGL2-scFv) were developed but were successful only in some brain tumor models. In the successful models, tumor cells injected intracranially into mice in remission were rejected via induction of tumor-specific T cells. Similarly, when T cells isolated from tumor-rejecting brains following FGL2-scFv cell treatment were transplanted into naïve mice, the intracranial tumor cell challenge was rejected. Of note, neither transplant donor mice nor recipient mice rejected a subcutaneous tumor cell challenge; only intracranial tumor cell challenge was rejected. These findings suggested the induction of brain T_RM_ cells, which was confirmed by *in vitro* phenotypic analysis and an *in vivo* CD103/CD69 antibody blocking study [16].

Despite this success, FGL2 scFv-T cell therapy only works in a limited number of tumor models. To develop a more effective therapy for preventing tumor relapse across multiple models, we investigated a subcutaneous Fgl2-KO tumor cell vaccine. Several whole tumor cell vaccine strategies have been tested. In some studies, tumor cells that were still tumorigenic were used, and tumors grew despite genetic engineering with immune-stimulatory genes. Therefore, the cells required irradiation before being used for vaccination [46]. In the present study, we discovered that Fgl2-KO tumor cells from the RT1 group lost the ability to propagate *in vivo* following subcutaneous inoculation, although the cells grew normally *in vitro*. Another distinctive aspect of our strategy is that it does not require insertion of an immune-stimulatory gene; only knockout or knockdown of the immune oncogene via either a viral or nonviral approach is needed. This is the first report of using oncogene deletion in whole tumor cell vaccination. In addition, to ensure the safety of the Fgl2-KO tumor cells, we also used a TK gene modification to deplete any leftover cells from the vaccine.

Skin vaccination using antigens of infectious pathogens is known to induce T_RM_ cells in the skin both locally and distantly, but it was not previously known whether a subcutaneous vaccination could induce T_RM_ cells in the brain. WCV has been widely investigated, but no prior study has tested the concept of T_RM_ induction in brains for rejecting tumor cell challenge. In addition, prior WCVs were not designed to prevent tumor relapse, which was our main goal in this study. Another limitation of the currently used WCVs is the overexpression of cytokines, which limits the use of high-dose vaccines due to toxicity concerns and a risk of promoting tumor growth and inducing immune suppression [47]. Third, the success rates of currently investigated WCVs for brain tumors have been limited. This Fgl2-CD47 circuit-disrupting WCV vaccine may be able to improve upon the success rates of other WCV. In summary, our report is the first to demonstrate that a cell-based vaccine can successfully induce brain T_RM_ cells to prevent tumor recurrence.

Our study further reveals that subcutaneous vaccination with Fgl2-KO tumor cells can transform naïve mice into brain tumor–rejecting mice across multiple tumor models and mouse strains (Figs. 1 and 2). Moreover, we showed that transplantation of BILs from vaccinated mice into naïve recipient mice results in tumor rejection in the recipients. In addition, phenotypic analysis confirmed induction of tumor-specific CD69^+^CD103+ T_RM_ cells, and depletion of T_RM_ cells with anti-CD103 or CD69 antibodies abolished tumor rejection (Figs. 3 and 4).

Despite our success in multiple tumor models and across mouse strains, some Fgl2-KO tumor cell lines (i.e., the RT2 group) failed to induce long-term survivors following intracranial tumor cell rechallenge. We reasoned that this failure was not attributable to the host mouse strain because different tumor cell lines derived from the same mouse strain had different efficacy (e.g., Fgl2-KO-4T1 and Fgl2-KO-DBT). Therefore, the key factor lies in the Fgl2-KO tumor cells themselves.

CD47, known as the “don’t eat me” signaling protein, is a key mediating factor. All the cell lines in which vaccination induced long-term survivors (i.e., the RT1 group) had low CD47 expression after Fgl2 KO, while the cell lines that did not produce an antitumor response (i.e., the RT2 group) maintained elevated CD47 expression levels, even in the wild-type cells. In the RT2 group, Fgl2 regulation on CD47 circuit appears to be disrupted. Across all RT2 models, Fgl2KO failed to reduce CD47 expression. This might be the key to understanding the WCV response and should be investigated further in the future. That said, we should also pay attention to the other known and unknown immune regulators produced by RT2 tumor cells such as B7.1 and MHCI, which may impact post-WCV T_RM_ induction and the efficacy of the WCV.

Overall, we demonstrated that CD47 plays an important role in RT1 cell lines. For example, the direct overexpression of CD47 in Fgl2-KO-DBT (RT1) cells reversed their intracranial tumor rejection capacity following subcutaneous vaccination (Fig. 6). Likewise, knocking down CD47 in wild-type DBT cells yielded the same effect as knockdown of Fgl2. These observations uncover the novel function of CD47 as an inhibitor in inducing T_RM_, although the exact mechanism remains unclear and will be elucidated in our future research. We speculate that CD47 may affect dendritic cells expansion, or antigen presentation by dendritic cells, which will be explored in future research. This direct CD47 knockdown strategy may be considered in our future WCV modification to circumvent the toxicity of systemic administering CD47 blockade, which may cause adverse effects such as anemia and platelet clearance in clinical trials [48]. This type of toxicity was not observed via our tumor-intrinsic CD47-knockdown strategy.

In summary, for the first time we found that a single-gene deletion from tumor cells can completely impair their tumorigenic property even though the tumorigenic mutations remain intact. Such impairment is due to the transformation of tumorigenic cells into tumor antigens. Compared to the tumorigenic whole tumor cell vaccine described in other studies [20,46,49], the present study provides a pathway to utilize live tumor cells to effectively induce long-term T_RM_ cells in brains for preventing brain metastases and primary brain tumor relapse via a single injection. Importantly, direct disruption of CD47 or both CD47 and Fgl2 may work not only against RT1 cells, but also RT2 cell lines, which will be a focus of our future efforts.

## Supplementary Material

1

## Appendix A. Supplementary data

Supplementary data to this article can be found online at https://doi.org/10.1016/j.canlet.2025.218215.

## LIST OF ABBREVIATIONS

Fgl2: Fibrinogen-like protein 2 gene
FGL2: Fibrinogen-like protein 2 protein
T_RM_: Tissue resident memory T cells
KO: Knockout
BIL: brain-infiltrating leukocyte
WCV: Whole tumor cell-based vaccine
WT: Wild type
CRISPR: Clustered Regularly Interspaced Short Palindromic Repeats
Cas9: CRISPR-associated protein 9
Src: Proto-oncogene tyrosine-protein kinase Src
PKCα: Protein Kinase C alpha
DC: dendritic cell
SCID: severe combined immunodeficiency
H&E: hematoxylin and eosin
TK: thymidine kinase
HSV: herpes simplex virus
RT1: response type 1
RT2: response type 2
Vac: Vaccination
CSFE: carboxyfluorescein succinimidyl ester
RPPA: Reverse Phase Protein Array
