# Supplementary material for: Fgl2-knockout tumor cells serve as a vaccine inducing long-duration brain-resident memory T cells that reject subsequent intracranial tumor cell challenges
Source: Cancer Lett. Author manuscript; Available in PMC 2026 Jul 13. (PMC13359019; doi:10.1016/j.canlet.2025.218215)
Supplement: 1 [file NIHMS2187901-supplement-1.docx]

**SUPPLEMENTARY FIGURE LEGENDS**


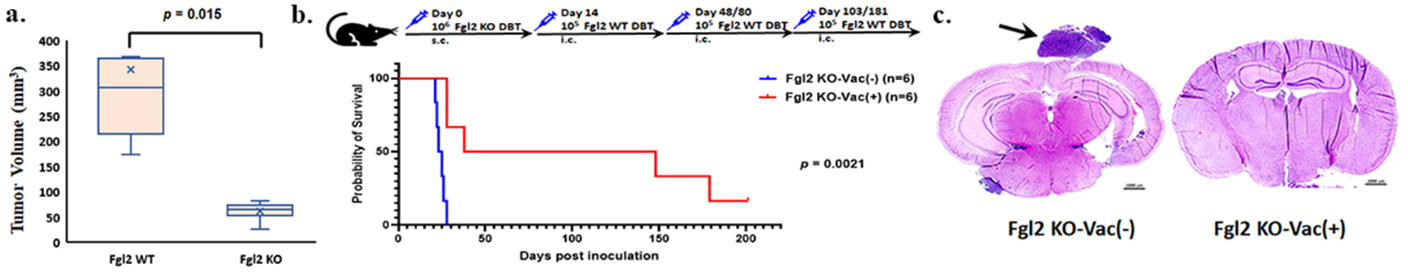


**Supplementary Figure 1. Vaccination with Fgl2 KO tumor cells protect mice from primary brain tumor development**

**a**. Comparison of tumor volumes post s.c. vaccination on day 10 from Fgl2WT DBT Vac (+) group vs. Fgl2 KO DBT Vac (+) group. **b**. Experiment scheme of the Fgl2 KO DBT whole cell vaccination (top) and Kaplan Meier survival curve following wildtype DBT tumor cell multiple challenges (bottom). Balb/c mice were subcutaneously vaccinated with 1x10^6^ Fgl2 KO DBT cells (Vac (+) group) or PBS (Vac (-) group) on day 0. Both groups received intracranial injections of 1x10^5^ wild type DBT cells on day 14, 48, and 103, and day 14, 80, and 181, respectively. Data was combined from two independent experiments with n=6 and 6 for Vac(+) (n=3, 3) and Vac(-) (n=3, 3), respectively. Log-rank test was performed to compare the survival difference of two groups (MST: Vac(-) 24.0 days; Vac(+) 93.0 days) . **c**. Comparison of H&E staining of intact mouse brain sections from Fgl2 KO GL261 Vac (+) group vs. Vac (-) group.


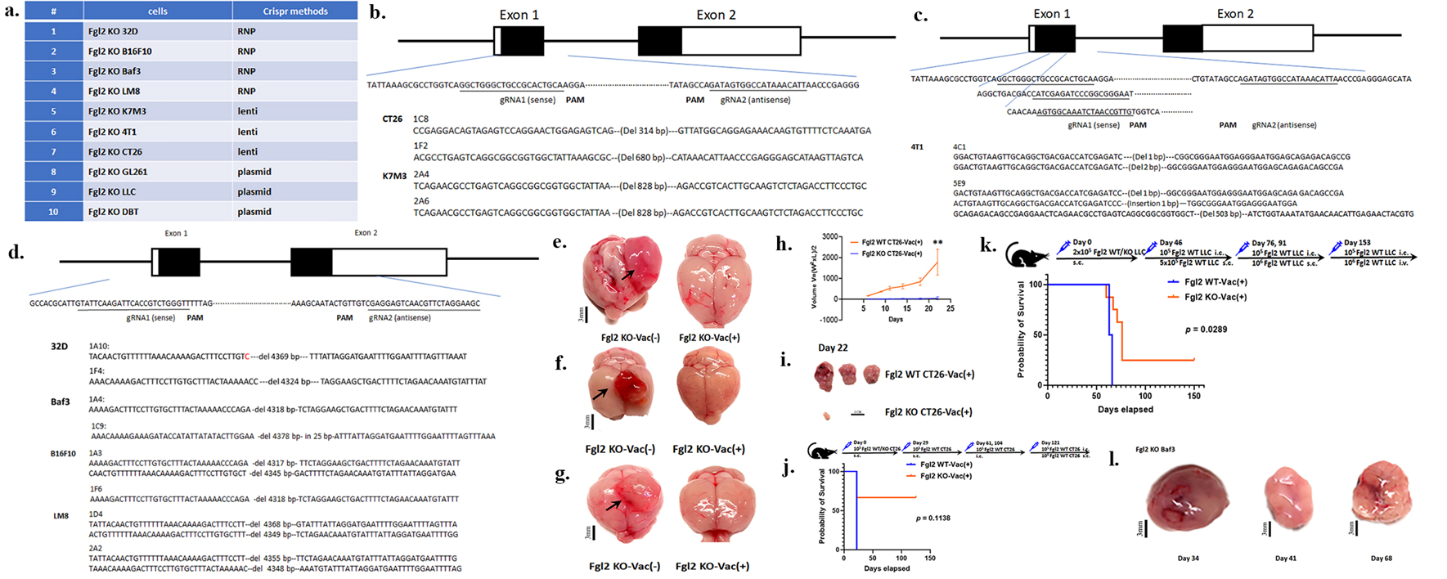


**Supplementary Figure 2.** **Vaccination with Fgl2 KO tumor cells protect mice from metastasis brain tumor development**

**a**. List of viral and non-viral CRISPR/CAS9 strategies to construct each Fgl2 KO cell line. RNP: Ribonucleoprotein (RNP) Delivery (directly deliver a ribonucleoprotein (RNP), consisting of the Cas9 protein in complex with a targeting gRNA, to the cells of interest). **b ~ d**. Targeted DNA fragment deletion in Fgl2 exon 1 or 2 in individual clones shown in each figure panel were validated by gene sequencing. Paired gRNAs were designed to excise exon 1 or exon 2 at the mouse Fgl2 locus. **e ~ g**. Representative whole brain pictures of Fgl2 KO CT26, K7M3, LLC Vac (+) group vs. Vac (-) group after intracranial challenge with wild type tumor cells on the day of sacrifice. **h**. Comparison of tumor growth curve post s.c. inoculation on day 6, 9, 11, 18, and 22 from Fgl2WT CT26 Vac (+) group vs. Fgl2 KO Vac (+) group. **i**. Representative subcutaneous tumor pictures of Fgl2WT CT26 Vac(+) group vs. Fgl2 KO Vac (+) group on day 22, respectively. **j**. Experiment scheme of the Fgl2 KO CT26 whole cell vaccination (top) and Kaplan Meier survival curve following wildtype CT26 tumor cell multiple challenges (bottom). Balb/c mice were subcutaneously vaccinated with 2x10^5^ Fgl2 KO CT26 cells (Vac (+) group, n=3) or PBS (Vac (-) group, n=3) on day 0. Tumor free mice from Fgl2 KO Vac(+) group received multiple challenges on day 29, 61, 104, and 121. For subcutaneous challenge, 1x10^5^ or 10^6^ wild type cells were injected via subcutaneous route; and for intracranial challenge, 1x10^5^ wild type cells were injected via intracranial route. Log-rank test was performed to compare the survival difference of two groups (MST: WT 22.0 days; KO undefined). **k**. Experiment scheme of the Fgl2 KO LLC whole cell vaccination (top) and Kaplan Meier survival curve following wildtype LLC tumor cell multiple challenges (bottom). C57BL mice were subcutaneously vaccinated with 2x10^5^ Fgl2 KO LLC cells (Vac (+) group, n=8) or PBS (Vac (-) group, n=2) on day 0. Tumor free mice from Fgl2 KO Vac(+) group received multiple challenges on day 46, 76, 91, 109, and 153. For subcutaneous challenge, 2x10^5^ or 1x10^6^ wild type cells were injected via subcutaneous route; for intracranial challenge, 1x10^5^ wild type cells were injected via intracranial route; and for intravenous challenge, 1x10^6^ wild type cells were injected via intravenous route. Log-rank test was performed to compare the survival difference of two groups (MST: WT 64.5 days; KO 76.0 days). **l**. Representative subcutaneous tumor pictures of BaF3 Fgl2 KO Vac (+) group on the day of sacrifice.


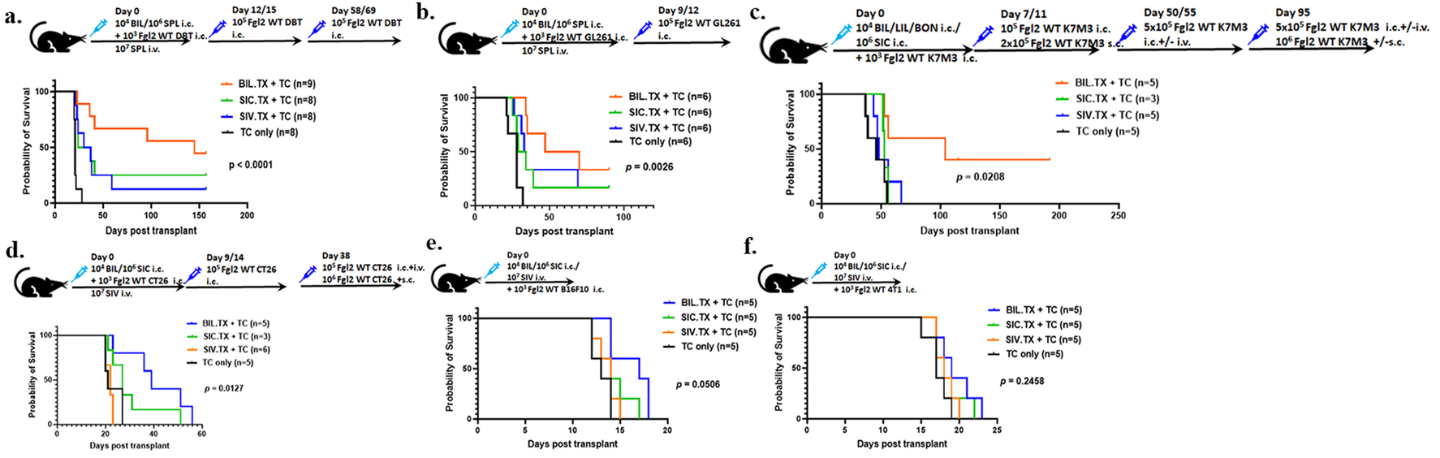


**Supplementary Fig 3 Adoptive transfer of the BIL and splenocytes from Fgl2 KO tumor cells vaccinated mice**

**a** ~ **e**. Experiment scheme (top) and Kaplan–Meier survival curves (bottom) of the BIL and splenocytes transplanted mice following wild type tumor cell challenge. The 1x10^6^ Fgl2 KO DBT, GL261, and K7M3 cells cells were inoculated on mice via subcutaneous route. After 10 days, the vaccinated mice were intracranially challenged with the cognate wild type tumor cells. Then the mice were euthanized, and the brains and spleen were collected on day 4. The BIL and splenocytes were harvest and enriched with CD45+ antibody-bound magnatic beads . The 1x10^4^ BIL with 1x10^3^ wild type tumor cells, 1x10^6^ splenocytes with 1x10^3^ wild type tumor cells, and/or 1x10^3^ wild type tumor cells were transferred into the brain of naïve mice. 1x10^7^ splenocytes were transferred into tail veins. On the indicated days, 1x10^5^ wild type tumor cells were intracranially challenged on transplanted mice. **a.** DBT cells (MST: TC 21.0 days; BIL 145.0 days; SIC 30.5 days; SIV 33.5 days), **b**. GL261 cells (MST: TC 28.0 days; BIL 58.5 days; SIC 31.5 days; SIV 33.5 days), **c**. K7M3 cells (MST: TC 46.0 days; BIL 104.0 days; SIC 53.0 days; SIV 48.0 days), **d**. CT26 (MST: TC 21.0 days; BIL 39.0 days; SIC 22.0 days; SIV 27.0 days), **e**. B16F10 (MST: TC 13.0 days; BIL 17.0 days; SIC 13.0 days; SIV 14.0 days), **f**. 4T1 (MST: TC 17.0 days; BIL 19.0 days; SIC 18.0 days; SIV 18.0 days).


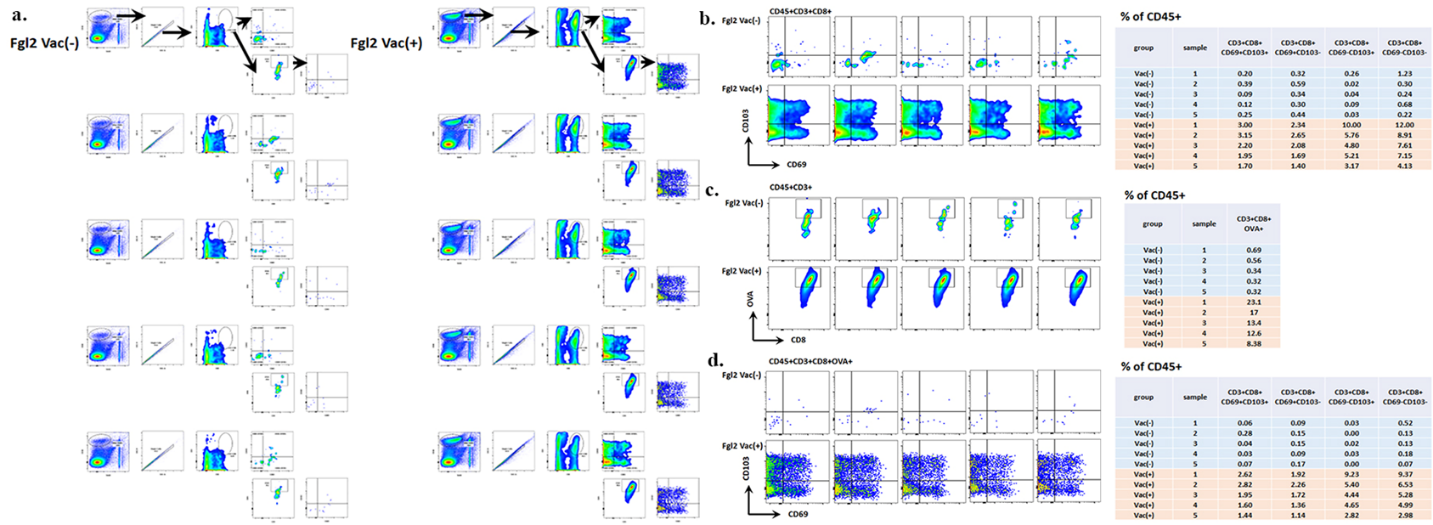


**Supplementary Figure 4. Gating strategy and result of flow cytometry-based phenotype analysis of the brain T_RM_ cells from the BIL of Fgl2 KO tumor cell vaccinated mice**

**a ~ d**. Brain-infiltrating leukocytes (BIL) from Vac(-) and Vac (+) vaccinated mice were analyzed for T_RM_ populations. 1x10^6^ Fgl2 KO GL261-OVA cells were vaccinated on mice via subcutaneous route. After 10 days, the vaccinated mice were intracranially challenged with wild type GL261-OVA cells. On day 4, the mice were euthanized, the brain were collected, and the BIL were harvested and stained with T_RM_ surface immune markers. **a**. The flow chart of lymphocytes gating. **b**. Phenotype result of each individual mouse (n=5) from both vaccinated (Vac(+)) and control mice (Vac(-)). For Vac(-) mice, only PBS was injected via subcutaneous route followed by intracranial wild type tumor cell challenge on day 10. Percentages of the T_RM_ subpopulations (CD69 vs CD103) of CD8^+^ T cells of the CD45^+^  BIL in the brain of the challenged mice were shown. **c**. Ova tetramer specific T_RM_ cell result from each individual mouse (n=5) of Vac(+) vs Vac(-). Percentages of OVA-specific CD8^+^ T cells in the CD45^+^ BIL in the brain of the challenged mice. **d**. Percentages of the T_RM_ subpopulations (CD69 vs CD103) of the OVA-specific CD8^+^ T cells in the CD45+ BIL in the brain of the challenged mice.


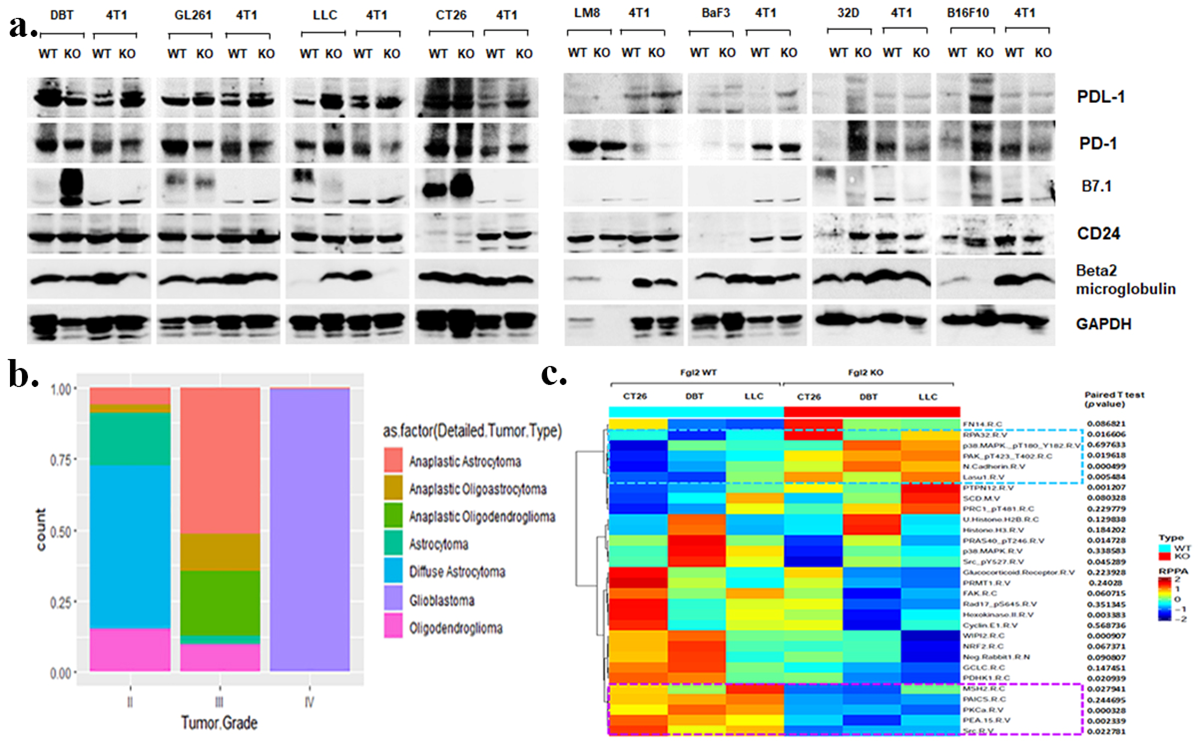


**Supplementary Figure 5. Impact of knocking out Fgl2 from tumor cells (Fgl2 KO tumor cells) on the expression of other key immune stimulators or checkpoints**

**a**. Expression levels of several key immune stimulatory or check point proteins were demonstrated by western blotting in the pairs of Fgl2 WT versus Fgl2 KO tumor cells. **b**. The stacked bar plot shows the percentage of different brain tumor types in each clinical grade. **c**. The proteomics analysis of DBT Fgl2-WT versus KO cells. The cell lysates were harvested and tested by RPPA platform. Data from RPPA were normalized to conduct differentiated analysis. The proteins with significant difference were filtered out and showed by the heatmap.

**Supplementary Table 1. Cell lines and source mouse strains**

| **Name** | **Description** | **Murine Strain Source** |
| --- | --- | --- |
| DBT | Delayed Brain Tumor | BALB/c |
| CT26 | Colon Carcinoma | BALB/c |
| K7M3 | Osteosarcoma | BALB/c |
| 4T1 | Mammary Carcinoma | BALB/c |
| GL261 | High-Grade Glioma/Glioblastoma | C57BL/6 |
| LLC | Lewis lung Carcinoma | C57BL/6 |
| B16F10 | Highly Metastatic Melanoma | C57BL/6 |
| LM8 | Osteosarcoma | C3H/HeJ |
| 32D | IL-3-dependent mast cell/ myeloid progenitor | C3H/HeJ |
| BaF | Ba/F3 IL‐3‐dependent, pro‐B cell | C3H/HeJ |

**Supplementary Table 2. Antibody information**

| **Target** | Label | Clone | **Source** | **Cat #** | **Dilutions** |
| --- | --- | --- | --- | --- | --- |
| CD103 | FITC | 2E7 | BioLegend | 121420 | 0.5μg/100μl |
| CD16/32  (FC block) |  |  | BD | 553141 |  |
| CD3 | APC/CY7 | 17A2 | Tonbo Biosciences | 25-0032-U100 | 0.5μg/100μl |
| CD4 | BV650 | GK1.5 | BioLegend | 100469 | 0.5μg/100μl |
| CD4 | R718 | H129.19 | BD | 752130 | 0.2μg/100μl |
| CD45 | BV570 | 30-F11 | BioLegend | 103135 | 0.5μg/100μl |
| CD8α | eFlor506 | 53-6.7 | Invitrogen | 69-0081-82 | 0.2μg/100μl |
| CD8α | BV650 | 53-6.7 | BioLegend | 100742 | 0.2μg/100μl |
| CD69 | Pacific Blue | H1.2F3 | BioLegend | 104524 | 0.5μg/100μl |
| OVA | PE | tetramer | NIH | 64685  63307 | 1ug/100ul |
| AH1 | PE | tetramer | MBL | TB-M521-1 | 10ul/100ul |
| CD47 |  | Polyclonal | Novus | AF1866 | 0.5ug/ml |
| PD-1 |  | Polyclonal | R&D | AF1021 | 0.2ug/ml |
| PD-L1 |  |  | BioX | BE0101 | 1:1000 |
| CD24 |  | Polyclonal | Novus | NBP1-46390SS | 1:1000 |
| B7.1 |  | Polyclonal | R&D | AF740 | 1ug/ml |
| Beta2 |  | 893803 | R&D | MAB8325 | 0.5ug/ml |
| GAPDH | HRP | 1E6D9 | Proteintech | HRP60004 | 1:1000 |
| Src |  | monoclonal | Cell Signaling | #2123 | 1:1000 |
| PKCa |  | Polyclonal | Cell Signaling | #2056 | 1:1000 |
